# Exosomes originating from infection with the cytoplasmic single-stranded RNA virus Rift Valley fever virus (RVFV) protect recipient cells by inducing RIG-I mediated IFN-B response that leads to activation of autophagy

**DOI:** 10.1186/s13578-021-00732-z

**Published:** 2021-12-25

**Authors:** Farhang Alem, Adeyemi A. Olanrewaju, Samson Omole, Heather E. Hobbs, Noor Ahsan, Graham Matulis, Christine A. Brantner, Weidong Zhou, Emanuel F. Petricoin, Lance A. Liotta, Massimo Caputi, Sina Bavari, Yuntao Wu, Fatah Kashanchi, Ramin M. Hakami

**Affiliations:** 1 School of Systems Biology, George Mason University, Manassas, VA USA; 2 Center for Infectious Disease Research (Formerly, National Center for Biodefense and Infectious Diseases), George Mason University, Manassas, VA USA; 3 Nanofabrication and Imaging Center, George Washington University, Washington, DC USA; 4 Center for Applied Proteomics and Molecular Medicine, George Mason University, Manassas, VA USA; 5 Charles E. Schmidt College of Medicine, Florida Atlantic University, Boca Raton, FL USA; 6 Healion Bio, Frederick, MD USA; 7 Present Address: Lentigen Technology, Inc., Gaithersburg, MD USA

**Keywords:** Exosome, Autophagy, IFN-B, RIG-I, Single-stranded RNA virus, Innate immune response, Viral RNA, Virus infection, Rift Valley fever virus, SARS-CoV-2

## Abstract

**Background:**

Although multiple studies have demonstrated a role for exosomes during virus infections, our understanding of the mechanisms by which exosome exchange regulates immune response during viral infections and affects viral pathogenesis is still in its infancy. In particular, very little is known for cytoplasmic single-stranded RNA viruses such as SARS-CoV-2 and Rift Valley fever virus (RVFV). We have used RVFV infection as a model for cytoplasmic single-stranded RNA viruses to address this gap in knowledge. RVFV is a highly pathogenic agent that causes RVF, a zoonotic disease for which no effective therapeutic or approved human vaccine exist.

**Results:**

We show here that exosomes released from cells infected with RVFV (designated as EXi-RVFV) serve a protective role for the host and provide a mechanistic model for these effects. Our results show that treatment of both naïve immune cells (U937 monocytes) and naïve non-immune cells (HSAECs) with EXi-RVFV induces a strong RIG-I dependent activation of IFN-B. We also demonstrate that this strong anti-viral response leads to activation of autophagy in treated cells and correlates with resistance to subsequent viral infection. Since we have shown that viral RNA genome is associated with EXi-RVFV, RIG-I activation might be mediated by the presence of packaged viral RNA sequences.

**Conclusions:**

Using RVFV infection as a model for cytoplasmic single-stranded RNA viruses, our results show a novel mechanism of host protection by exosomes released from infected cells (EXi) whereby the EXi activate RIG-I to induce IFN-dependent activation of autophagy in naïve recipient cells including monocytes. Because monocytes serve as reservoirs for RVFV replication, this EXi-RVFV-induced activation of autophagy in monocytes may work to slow down or halt viral dissemination in the infected organism. These findings offer novel mechanistic insights that may aid in future development of effective vaccines or therapeutics, and that may be applicable for a better molecular understanding of how exosome release regulates innate immune response to other cytoplasmic single-stranded RNA viruses.

**Supplementary Information:**

The online version contains supplementary material available at 10.1186/s13578-021-00732-z.

## Introduction

Rift Valley Fever (RVF) is an arthropod-borne zoonotic disease responsible for epidemics in Africa and the Arabian Peninsula. The causative agent, Rift Valley Fever virus (RVFV; *Bunyavirales: Phenuiviridae*), primarily utilizes a mosquito vector to infect both humans and ruminants [1]. RVF causes near 100% abortion rates in pregnant ruminants and can be fatal in humans [2]. Symptoms may vary from a mild, flu-like syndrome to ocular, encephalitic, or hemorrhagic syndromes, with a case fatality rate in humans that is normally less than 1% but that can reach greater than 30% in isolated outbreaks [3]. Mortality estimates associated with the most notable RVF epizootics are 100,000 animal deaths and 600 human fatalities [3]. Considering the high economic and public health impacts of RVFV, NIAID has classified RVFV as a pathogen of highest concern (Category A). RVFV is considered to have a high colonization capacity due to its ability of surviving a wide range of environments, raising strong concerns over the potential for its introduction and establishment in western regions [4].

RVFV is an enveloped virus, with a 12 Kb viral genome that comprises three single stranded RNA segments: L, M, and S. The L and M segments are of negative polarity and the S segment is of ambisense polarity [5]. The L segment encodes the viral RNA dependent RNA polymerase (RdRp), which is packaged into virions and synthesizes initial RVFV transcripts [6, 7]. The M segment encodes a polyprotein precursor that is later cleaved into the viral envelope proteins Gn, Gc, and the accessory proteins NSm, and P78/NSm-Gn [8, 9]. The S segment encodes the nucleocapsid (N) protein and the nonstructural protein NSs that serves as a critical virulence factor modulating multiple cellular pathways. NSs suppresses cellular mRNA transcription by sequestering the p44 subunit of the transcription factor II (TFIIH), and by triggering the degradation of the p62 subunit [10, 11]. Furthermore, NSs helps evasion of the host innate immune response by two independent mechanisms: interferon beta (IFN-B) inhibition, and degradation of a double-stranded RNA-dependent protein kinase (EIF2AK2). NSs inhibits the synthesis of IFN-B mRNA by forming a multiprotein complex with Sin3A-associated protein 30 (SAP30) and the Yin Yang 1 (YY1) transcription factor, preventing activation of the IFN-B promoter by factors such as NF-кB and activator protein 1 (AP1) [12–14]. NSs degradation of EIF2AK2 relieves viral repression, as EIF2AK2 mediates antiviral activity through translation arrest of cellular and viral mRNA [15]. Overall, NSs prevents an antiviral response by halting the transcription of host mRNA and preventing a shut-off of translation in order to maintain viral protein synthesis [16].

Exosomes are a subclass of extracellular vesicles (EVs) that have gained attention due to their role in human disease progression, including modulation of the immune response to viral and bacterial infections [17–19]. Exosomes are characterized by a diameter of 30–150 nm, specific density range, and the presence of several exosome markers such as CD63, CD81, CD9, TSG101, and Alix [17, 20, 21]. Their cargo consists of various RNA species, proteins, and lipids, and varies depending on several factors such as cellular origin or type of infection [22–25]. Exosomes originate from the inward budding of a late stage endosome (multivesicular endosomes, or MVEs for short) [26]. The fusion of the multivesicular endosomes with the plasma membrane at the cell surface allows the secretion of exosomes into the extracellular environment [27].

Although the importance of exosomes and other EVs in modulating immune response during viral infections has been recently demonstrated by us and others [17, 20, 28–30], their contribution to the replication and pathogenesis of RNA viruses remains largely unexplored. In particular, a significant gap in knowledge exists for cytoplasmic single-stranded RNA viruses such as SARS-CoV-2 and RVFV. In a previous study, we isolated and characterized exosomes from RVFV-infected cell clones that showed resistance to RVFV-induced cell death (called resistant clones) and demonstrated that they carry signal for the viral RNA genome and may be associated with specific viral proteins [28]. However, the mechanisms of how these exosomes may affect viral production in the recipient cells to influence the infection process were not investigated. In this study, we optimized a purification procedure involving density gradient separation for the isolation of exosome preparations, and investigated whether and how exosomes purified from RVFV-infected cells (EXi-RVFV) regulate antiviral responses in proximal or distal recipient cells, thus affecting disease progression. This effort also allowed comparing the EXi-RVFV with the resistant clone exosomes in regard to associated viral proteins as some of the isolated clones showed varied profiles. We show here that density gradient purified EXi-RVFV carry viral genomic segments and selectively package a specific viral protein cargo (N and Gc/Gn proteins). We also demonstrate that they serve a protective role for the host by priming naïve recipient cells to inhibit viral replication in the event of becoming infected with RVFV. Furthermore, we show that the mechanism for this protection is EXi-RVFV induction of IFN-B in the recipient cells, through the activation of the RIG-I pathway, that leads to subsequent activation of autophagy. These results suggest a model in which viral RNA genome segments carried by the EXi-RVFV boost the host response to RVFV infection by RIG-I-dependent activation of the IFN pathway, enabling naïve recipient cells to fight off infection upon encountering RVFV through increased autophagic activity. As the importance of type I IFNs and autophagy modulation have been demonstrated for infections involving other cytoplasmic single-stranded RNA viruses (e.g., COVID-19), these findings warrant future analysis of whether similar host response mechanisms involving exosomes also occur during such infections.

## Materials and methods

### Cell culture and reagents

Vero (African green monkey kidney) cells (ATCC, CCL-81) were maintained in Dulbecco’s modified minimum essential medium (DMEM) supplemented with 10% heat inactivated Fetal Bovine Serum (FBS), 1% l-Glutamine, and 1% penicillin/streptomycin. U937 monocytes (same source as in reference 28) were grown in RPMI-1640 (Roswell Park Memorial Institute) supplemented with 10% FBS, 1% l-Glutamine, and 1% penicillin/streptomycin. Human Small Airway Epithelial Cells (HSAECs) were obtained from Cambrex Inc., Walkersville, MD, and maintained in DMEM supplemented with 10% FBS, 1% l-Glutamine, and 1% penicillin/streptomycin. All cell lines were maintained at 37 °C in 5% CO_2_. To prepare exosome-free media (EFM), FBS was subjected to ultracentrifugation at 40,000 rpm (Beckman Type 70Ti rotor) for 3 h in an FBS:media ratio (v/v) of 1:1.

### Exosome isolation and purification

Sterile solutions and supplies were used throughout the exosome isolation and purification procedures. Vero cells were expanded into two T-225 flasks in EFM and were either mock-infected or infected at MOI 1 with double-tagged MP12-L-V5, NSs-Flag RVFV (kind gift of Dr. Shinji Makino, UT Galveston, Galveston, TX, USA) for 1 h at 37 °C, 5% CO_2_. The inoculum was then removed and cells were washed once with PBS prior to addition of 100 ml of EFM. Culture supernatants were collected 36 h post infection (p.i.), a time point at which the full viability of the infected cells was quantifiably verified (Additional file 1: Figure S1), using both the CellTiter-Glo® Luminescent Cell Viability Assay (Promega) and Luna-FL cell counter measurements (Logos Biosystems) using acridine orange/propidium iodide (AO/PI) staining. The culture supernatants were centrifuged at 2000 rpm for 10 min at 4 °C to eliminate dead cells. Supernatants were then passed through 0.22 µm filter, followed by centrifugation at 10,000 x *g* for 30 min at 4 °C. The resulting supernatants were then subjected to ultracentrifugation at 117,000 x g (Beckman Type 70Ti rotor) for 2 h at 4 °C. The supernatants were discarded and exosome pellets were resuspended in PBS without calcium and magnesium and subjected to another round of ultracentrifugation at 117,000 x *g* for 2 h at 4 °C. Exosome pellets were then resuspended in sterile PBS without calcium and magnesium containing 1 × Halt Protease Inhibitor Cocktail (Thermo Fisher Scientific), and subjected to sucrose density gradient centrifugation. For the density gradient purification, a 2 M sucrose solution stock was used to prepare five sucrose fractions with the following densities, using PBS as a diluent: 2 M, 1.18 M, 0.91 M, 0.67 M, and 0.37 M. A step gradient was created by using two milliliters of each fraction to form density layers inside a Beckman centrifuge tube (Item no. 331372), starting with addition of the densest fraction to the bottom of the tube and carefully layering over the next most dense layer in succession to create the gradient. The semi-purified exosome samples were layered on top of the gradient followed by ultracentrifugation at 200,000 x g for three hours at 4 °C. Taking into account the volume of the layered exosome sample, each fraction was removed and the 1.08 g/ml and 1.12 g/ml density fractions, which were among the three fractions that contained exosomes based on CD63, TSG101, and Flotilin-1 western analyses, were diluted by addition of 20 ml of PBS and subjected to ultracentrifugation at 117,000 x g for two hours to remove residual sucrose. The resulting gradient-purified exosome pellets were then resuspended in PBS containing 1 × Halt Protease Inhibitor Cocktail, were combined and filter sterilized using a 0.22 µm syringe filter (VWR), and subsequently stored at −80 °C. The protein concentrations of exosome preparations were determined using the BCA protein assay kit (Pierce), and plaque assay of the purified exosomes was performed as described below to verify that they are completely free of virions. For all the studies reported here, these sucrose gradient-purified EXi-RVFV and EXu (control) preparations were used. At least 20 biological replicates (fully independent runs) of exosome purifications and characterizations were performed in the course of our studies.

### Plaque assays

Vero cells were seeded in a six well plate at a density of 1 × 10^6^ cells per well and incubated for 24 h to allow reaching 90%–100% confluency. Culture supernatant containing RVFV MP12 strain was serially diluted tenfold in DMEM from 10^–1^ to 10^–7^, and 400 µL of diluted inoculums were used to infect each well in duplicates. Vero cells were infected for 1 h, followed by a 3 ml overlay of a 1:1 mixture of 0.6% agarose in deionized H_2_O (diH_2_O) and 2X EMEM supplemented with 5% FBS, 2% penicillin/streptomycin, 1% l-Glutamine, 1% Sodium Pyruvate, and 1% nonessential amino acids. The overlay was allowed to solidify at room temperature and incubated for 72 h. 10% formaldehyde in diH_2_O was used to fix the cells for 1 h at room temperature. The agarose plugs were gently removed, and the cells were stained using 1% crystal violet, 20% methanol in diH_2_O solution for 30 min at room temperature. Average number of plaques were enumerated for each treatment, and the following equation was used to deduce viral titer: pfu/ml = average number of plaques x dilution factor × 2.5.

### ZetaView analysis

Nanoparticle tracking analysis was performed using the ZetaView Z-NTA (Particle Metrix) and its corresponding software (ZetaView 8.04.02). The machine was calibrated according to manufacturer’s protocol. Briefly, 100 nm polystyrene nanostandard particles (Applied Microspheres) were used to calibrate the instrument prior to sample readings at a sensitivity of 65 and a minimum brightness of 20. Automated quality control measurements including, but not limited to, cell quality check and instrument alignment and focus were also performed prior to use. For each measurement, the instrument pre-acquisition parameters were set to a temperature of 23 °C, a sensitivity of 85, a frame rate of 30 frames per second (fps), and a shutter speed of 250. For each sample, 1 ml of the sample diluted in diH_2_O, was loaded into the cell, and the instrument measured each sample at 11 different positions throughout the cell, with three cycles of readings at each position. After automated analysis and removal of any outliers from the 11 positions, the mean size (indicated as diameter), the concentration of the sample, and the Zeta potential value, were calculated. Measurement data from the ZetaView were analyzed using the corresponding software, ZetaView 8.04.02, and Microsoft Excel 2016. For each sample, three biological replicates were analyzed.

### Transmission electron microscopy

Various dilutions of the exosome samples were prepared and 5 μl was adsorbed to the formvar side of a glow-discharged, 200 mesh copper grids with formvar and carbon coating. Excess sample was removed from the grids with filter paper. Samples were fixed with 4% glutaraldehyde in 0.12 M sodium cacodylate buffer, pH 7.2, for 5 min. The glutaraldehyde was then removed with filter paper and 4 washes were performed with distilled, deionized water. Grids were stained with 1% aqueous uranyl acetate for 60 s. The uranyl acetate was then removed with filter paper and grids were allowed to air dry. Images were captured using an FEI Talos F200X transmission electron microscope at 80 kV (Thermo Fisher Scientific, Hillsboro, OR).

### Mass spectrometry analysis

Density gradient-purified exosomes (EXu and EXi-RVFV) were dissolved in 8 M urea containing 1X Halt Protease Inhibitor Cocktail. Proteins were reduced by treatment with 10 mM dithiothreitol (DTT) for 15 min at 37 °C, alkylated by treating with 50 mM iodoacetamide for 10 min at room temperature, and digested by incubation with trypsin for 4 h. The tryptic peptides were analyzed by high sensitive nanospray liquid chromatography-coupled tandem mass spectrometry (LC–MS/MS) using an Orbitrap Fusion mass spectrometer (Thermo Fisher Scientific). After sample injection, peptides were eluted using a linear gradient of 5% mobile phase B (0.1% formic acid, 80% acetonitrile) to 50% B in 60 min at 300 nL/minute, then to 100% B in an additional 5 min. The Orbitrap Fusion was operated in a data-dependent mode in which each full MS scan (100,000 resolving power) was followed by multiple MS/MS scans at fastest speed. Tandem mass spectra were searched against the Chlorocebus sabaeus and Rift Valley Fever virus protein database using Proteome Discoverer (version 2.1) with full tryptic cleavage constraints, static cysteine alkylation by iodoacetamide, and variable methionine oxidation. Mass tolerance for precursor ions was 3 ppm and mass tolerance for fragment ions was 0.5 Da. The search results were filtered by high stringent criteria with 1% false discovery rate. Analysis was performed in biological duplicates.

### Western blot analysis

Total protein levels in whole cell lysates were quantified using the BCA protein assay kit (Pierce). Cell lysates matched for total protein levels based on BCA results (30 µl total volume) were heated at 70 °C for 10 min before separation on NuPAGE 4–12% Bis–Tris gels (Novex). Protein transfer was performed either using the iBlot gel transfer nitrocellulose stacks (Novex), or by wet transfer using Immobilon PVDF membrane (Millipore). Membranes were blocked in 5% milk in TBS containing 0.1% tween-20 (TBS-T) and then incubated overnight with the appropriate antibody: α-CD63 (Abcam ab8219, 1:500); α-TSG101 (BD Biosciences 612696, 1:1000); α-Flotillin-1 (Cell Signaling Technology #18634, 1:1000); α-Gn protein (USAMRIID hybridoma facility, Ft. Detrick, MD, 1:1000; also see reference #38); α-N protein (generous gift of Dr. C. Schmaljohn-USAMRIID, 1:500); α-V5 (Serotec MCA1360, 1:1000); α-Flag (Sigma-Aldrich F1804, 1:1000); α-IFN-B (Cell Signaling 73671, 1:1000); α-CD9 (Santa Cruz sc-13118, 1:200); α-RIG-I (Santa Cruz sc-48929, 1:1000); α-LC3B (Sigma; L7543), and, α-beta actin (Abcam ab49900, 1:5000). The membranes were then washed to remove excess primary antibody and incubated with the appropriate secondary HRP-conjugated antibody. Subsequently, the membranes were washed again to remove excess secondary antibody and the protein bands were visualized by Super Signal West Femto Maximum Sensitivity Substrate kit (Thermo Scientific) using a ChemiDoc XRS System (Bio-Rad).

### RNA isolation and quantitative RT-PCR (RT-qPCR) analysis of viral genome

Total RNA from different samples was isolated using the trizol-chloroform method. Approximately 400 ng of isolated RNA was used for cDNA synthesis with GoScript Reverse Transcriptase System (Promega) using Random Primers. For RT-qPCR, primer pairs used for each of the three segments were as follows: A) L segment: RVFV L Polymerase Forward (5′-GGT GGC ATG TTC AAT CCT TT-3′; Tm = 54 °C), and RVFV L Polymerase Reverse (5′-GCA TTC TGG GAA GTT CTG GA-3′; Tm = 54 °C); B) M segment: Gn Forward (5′-AAA GGA ACA ATG GAC TCT GGT CA-3′, Tm = 58 °C), and Gn Reverse (5′-CAC TTC TTA CTA CCA TGT CCT CCA AT-3′; Tm = 58 °C); C) S segment: RVFV NSs Forward (5′-TCT GAA AGA AGC CAT ATC CT-3′; Tm = 54 °C), and RVFV NSs Reverse (5′-CTC GCT ATC ATC CTG TGT AA-3′; Tm = 54 °C); and, also, RVFV N Forward (5′-CAT GGT GGA TCC TTC TCT AC-3′; Tm = 54 °C) and RVFV N Reverse (5′-CTA TTC ACT GCT GCA TTC AT-3′; Tm = 54 °C). The PCR cycling conditions consisted of 1 cycle at 95 °C for 2.5 min, 39 cycles at 95 °C for 15 s, and 58 °C (for L and Gn primer sets) or 54 °C (for N and NSs primer sets) for 40 s. The RNA count of the samples was determined relative to the reference gene GAPDH, or was based on the cycle threshold (Ct) value relative to the standard curve. The dilutions of plasmids containing sequences of the segments of RVFV genome cDNA were used as quantitative standards**.** Every assay was performed in technical triplicates and biological triplicates.

### Cell viability and growth assays

Approximately 2.5 × 10^5^ U937 cells, or human small airway epithelial cells (HSAECs), were seeded in 1 ml of exosome free RPMI per well of a 24 well plate. Cells were then treated with 5 µg of purified exosome samples (corresponding to 50 vesicles per cell) and allowed to incubate. At 0 h, 24 h, and 48 h post treatment, each well was thoroughly mixed and an aliquot was removed for analysis. Each aliquot was mixed with the acridine orange/propidium iodide (AO/PI) stain in a 1:9 ratio and sample fluorescence was quantified using a Luna-FL cell counter (Logos Biosystems). Assays were performed in technical duplicates and biological triplicates.

### Caspase 3 assay

A positive control for the assay was generated by exposing U937 cells to 10 Gy of ionizing irradiation and then culturing the cells for 72 h and preparing whole cell lysate for analysis. We have verified that this treatment condition induces apoptotic pathways, as measured by caspase 3 activation, without causing any significant change in cell viability. For each treatment condition, a total of 1 × 10^6^ U937 cells was used. Untreated cells, irradiated cells, and cells treated with either 20 µg density gradient-purified EXu or EXi-RVFV (i.e., 50 vesicles per cell) were analyzed at 48 h post treatment using the Caspase 3 Colorimetric Kit (BioVision), following the manufacturer’s instructions. Briefly, approximately 200,000 U937 cells were resuspended in caspase 3 sample lysis buffer at 4 °C for 10 min, and the homogenates were then centrifuged at 10,000 × g and the supernatants were quantified using the BCA protein assay kit (Pierce). A volume containing 100 µg of total protein was diluted appropriately with cell lysis buffer, and then diluted 1:1 with reaction buffer containing 10 mM DTT. DEVD-pNA was then added at a final concentration of 200 μM and incubated for 1 h at 37 °C. Samples were further diluted to 1 ml with dilution buffer, and absorbance was measured at 400 nm using the Thermo Scientific Genesys 20 spectrophotometer. Assays were performed in technical duplicates and biological triplicates.

### Caspase 1 assay

Active recombinant caspase-1 (Biovision) was used as positive control. Samples were analyzed using the Caspase 1 Colorimetric Kit (BioVision), following the manufacturer’s instructions. The samples analyzed, and the methodology used, were identical to the caspase 3 assay, except that YVAD-pNA was used as substrate. Assays were performed in technical duplicates and biological triplicates.

### Viral uptake and replication analysis

5 × 10^5^ U937 cells, or HSAECs, were either left untreated or were treated with 10 μg of density gradient-purified EXu or EXi-RVFV for 24 h and subsequently infected with MP12-L-V5, NSs-Flag RVFV (MOI 1) for 1 h at 37 °C, 5% CO_2_. The inoculum was then removed, and the cells were washed twice with PBS and either lysed immediately (for entry assay) or incubated back in culture medium and lysed at either 6 h or 24 h post infection (to analyze replication effects). Plaque assays were performed to determine the viral load in the lysates. Assays were performed in technical duplicates and biological triplicates.

### IFN-B assays

For western blot analysis, 5 × 10^5^ U937 cells, or HSAECs, were either left untreated or were treated with 10 μg of density gradient-purified EXu, or EXi-RVFV, or were infected with either the doubly tagged MP12-L-V5, NSs-Flag strain of RVFV or with the RVFV ΔNSs strain at MOI of 0.1. At 24 h post treatment/infection, cells were lysed in PBS containing 0.1% Triton X-100 and 1X Halt Protease Inhibitor Cocktail (Thermo Fisher Scientific), and total protein was quantified by BCA (Pierce). A total of 10 μg of protein was run on a gel and analyzed by western for IFN-B expression. Assays were performed in biological triplicates.

For the RT-qPCR analysis to quantify IFN-B levels, in addition to the same exosome treatments used for the western blot assays, treatment with Poly(I:C) was also included to serve as a positive control for induction of IFN-B expression. U937 cells were collected 12 h post treatment and washed with 1X PBS, lysed with TRI Reagent (Zymo Research), and their intracellular RNA was extracted using Direct-zol Miniprep RNA kit (Zymo Research, R2053) per manufacturer’s instructions. Briefly, equal volume of 100% ethanol was added to the sample lysed in TRI Reagent and vortexed for 5 s. The mixture was transferred into a Zymo-Spin IICR Column in a collection tube and centrifuged for 30 s at 10,000 × *g*. The column was transferred to a new collection tube and 400 μl of RNA Wash Buffer was added to the column and centrifuged for 30 s at 10,000 × *g*. After centrifugation, 80 μl of DNase I was added to the column and allowed to incubate at room temperature for 15 min. After incubation, 400 μl of Direct-zol RNA PreWash was added to the column and centrifuged for 30 s at 10,000 × *g* and this step was repeated. After the second wash, 700 μl of RNA Wash Buffer was then added to the column and it was centrifuged for 1 min at 10,000 × *g*. The column was transferred into RNase-free tube and 25 μl of DNase/RNase-Free Water was added directly to the column and centrifuged for 30 s at 10,000 × *g* to elute the RNA for RT-qPCR analysis. RNA concentrations were determined using a NanoDrop One spectrophotometer (Thermo Scientific), and equal amounts were used with the Taqman RNA to Ct One Step Kit (Thermo Scientific, 4392938) using an IFN-B primer probe kit (Thermo Scientific, Hs02621180_S1). All procedures were performed according to the manufacturer’s recommended procedures, and RNA concentrations were determined using the ∆∆Ct method, normalizing to the 18 s housekeeping gene. The RT-qPCR assay was performed in biological triplicates.

### Immunoprecipitation studies

Density gradient-purified EXi-RVFV were incubated with Protein A/G magnetic Dynabeads (Thermo Fisher Scientific 88802) coated with human anti-CD9 antibody (Thermo Fisher Scientific MA1-80307) at room temperature for 1 h. The beads were then pulled down using a magnetic stand, washed two times with PBS, and resuspended in SDS loading buffer for analysis by immunoblotting using anti-CD9 antibody. The starting material, as well as the EXi-depleted supernatants (ΔEXi), were also compared for the ability to induce IFN-B expression in recipient cells, using both western blot and RT-qPCR assays. For the western blot analysis, lysates from U937 cells that were treated with EXi-depleted IP supernatant were analyzed, and whole cell lysates were also included from uninfected U937 cells, U937 cells infected with either the MP12 strain or ∆NSs strain of RVFV, and U937 cells treated with either density gradient-purified EXu or EXi-RVFV. The RT-qPCR analysis was performed as described above in “Methods” section for “IFN-B Assays”. All assays were performed in biological triplicates.

### RIG-I siRNA knockdown

U937 cells (5 × 10^5^ per sample) were transfected with either RIG-I siRNA (Santa Cruz) or Control siRNA-I (Santa Cruz). Transfection reagent (FuGENE^®^ 6, Promega) was mixed with siRNA at a ratio of 4:1 in Opti-MEM media, and incubated at room temperature for 15 min. The mixture was then added to the cells and incubated for 24 h. Subsequently, the cells were either left untreated, or were treated with 10 μg of density gradient-purified EXu or EXi-RVFV, or were infected with either MP12-L-V5, NSs-Flag strain of RVFV or the RVFV ΔNSs strain at MOI 0.1. At 24 h post treatment/infection, cells were lysed in 1 × PBS containing 0.1% TX-100 and 1X Halt Protease Inhibitor Cocktail (Thermo Fisher Scientific), and total protein was quantified by BCA (Pierce). A total of 10 μg of protein was run on a gel and analyzed by western for IFN-B expression. Assays were performed in biological triplicates.

### RNase assay

An amount of EXi-RVFV or EXu corresponding to 10 µg of total protein was resuspended in 50 µl of PBS. RNase A was subsequently added to the exosome samples at a final concentration of 100 μg/ml. As control, EXu and EXi-RVFV without RNase A treatment were also included side by side and processed identically for analysis. RNase-treated samples and control samples were incubated for 1 h at 37 °C. Subsequently, the samples were dialyzed against PBS using Slide-A-Lyzer Dialysis Cassettes 20 K MWCO (Thermo Fisher Scientific) to remove the RNase A. ZetaView analysis of the samples was performed to verify that RNase treatment has not affected the integrity of the vesicles. To test for activity, a total of 5 × 10^5^ U937 cells were either left untreated or were treated with EXi-RVFV + RNase, EXi-RVFV, or EXu. At 24 h post treatment, cells were lysed in PBS containing 0.1% Triton X-100 and 1X Halt Protease Inhibitor Cocktail (Thermo Fisher Scientific), and total protein was quantified by BCA (Pierce). A total of 10 μg of protein was used for western blot analysis for IFN-B expression, as described in the western blot analysis section of the methods. Assays were performed in biological triplicates.

### Interferon inhibition assay

U937 cells were pretreated for 4 h with 5 μg/ml of IFNAR1/2 antibody (anti-Interferon-A/B Receptor Chain 2) from Millipore (cat# MAB1155). Subsequently, the cells were either left untreated or were treated with EXi-RVFV or EXu at a concentration of 50 particles per cell. Cells were then harvested after 24 h, lysed in RIPA buffer, and treated with 1X Halt protease inhibitor Cocktail (Thermo Fisher Scientific). Autophagy levels were evaluated by LC3 western blot analysis of whole cell lysates matched for total protein levels, as described in the western blot analysis of the materials and methods section.

### LC3 immunofluorescence staining

Briefly, 1 x 10^6^ U937 cells were suspended in 1 ml complete RPMI media in a 12 well plate and either were left untreated, or were treated with either EXi-RVFV or EXu for 24 h. Subsequently, the contents of each well were spun down, and the cells were fixed by treating with 4% paraformaldehyde for 20 min at room temperature. The cells were then washed twice with PBS buffer and permeabilized by treatment with 0.1% Triton X-100 in PBS for 5 min. The cells were subsequently washed twice with PBS and blocked with 5% bovine serum albumin in TBS containing 0.1% Tween-20 (TBS-T) for 1 h. Next, the cells were incubated with LC3 primary antibody specific for immunofluorescence diluted 1:100 in PBS for 30 min at room temperature, followed by 2 washes with PBS. The cells were then incubated with Alexa Fluor secondary antibody (Invitrogen, A11034) in the dark for 30 min at room temperature, followed by 2 washes with PBS. They were then resuspended in 30 µl of Fluoromount-G mounting medium with DAPI and analyzed using Nikon Eclipse Ti2 fluorescent microscope. For each sample, twenty distinct fields of cells were examined, corresponding to at least 200 cells per treatment.

### Statistical analysis

Statistical analysis was performed using R studio version 0.99.465, unless otherwise noted. Normality and homogeneity of variance were tested with Shapiro–Wilk and Barlett’s tests. For parametric data, significance was tested using either one-way ANOVA with Dunnett’s post-hoc or the student’s t-test. For non-parametric data, significance was tested using Kruskal–Wallis with Dunn’s post-hoc test.

## Results

### Purification and characterization of exosomes

**Fig. 1:**
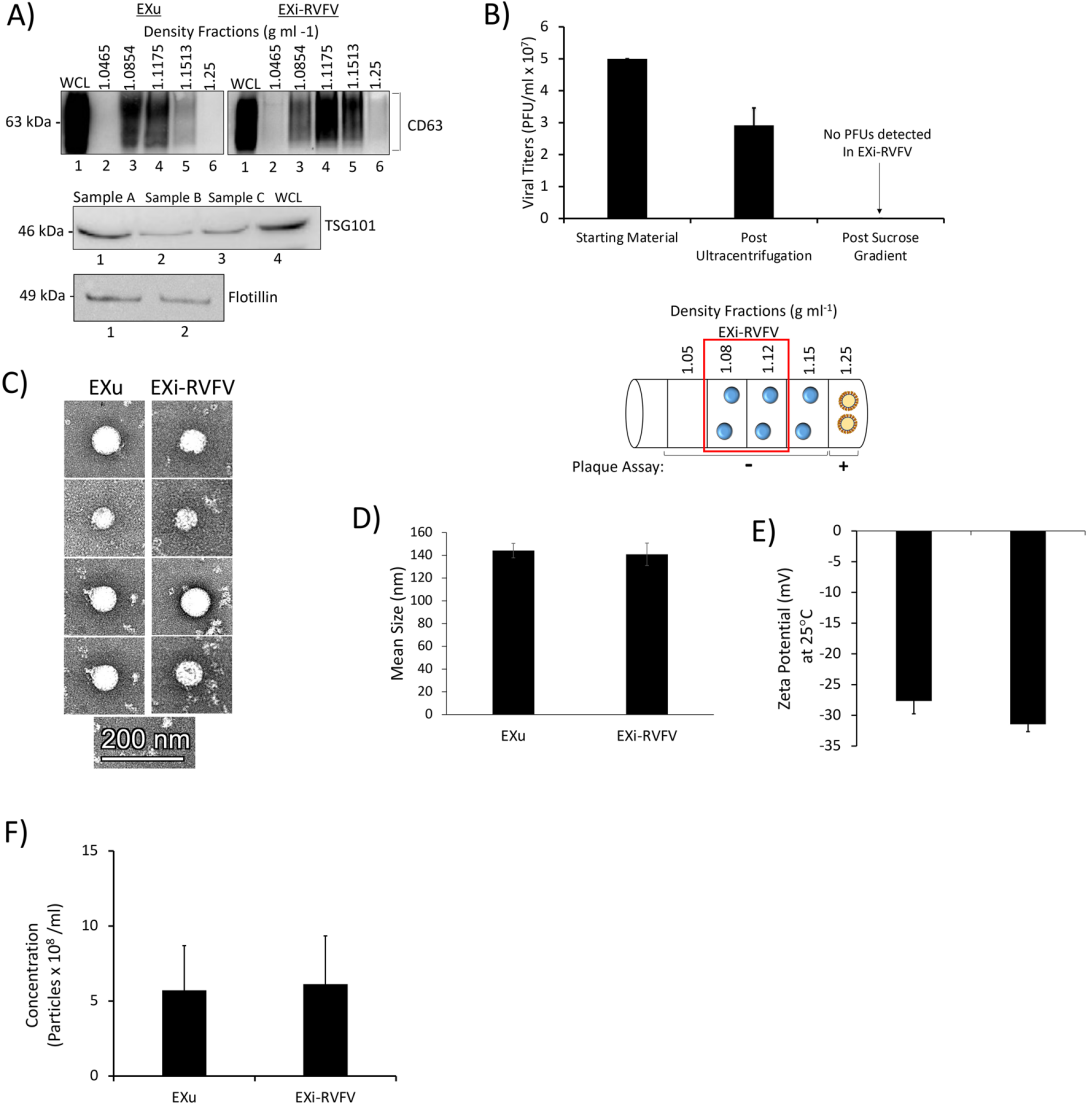
Characterization of Sucrose Gradient-Purified Exosomes.** A** Exosomes from both RVFV-infected cells and uninfected cells were harvested in parallel using the procedure describe in the Materials and Methods section. The sucrose density fractions were initially analyzed by western blot for the exosome marker CD63 (top). CD63-positive fractions with densities 1.08 g ml^-1^ and 1.12 g ml^-1^ were combined and further analyzed by western blot for additional exosome markers TSG101 (middle) and Flotillin-1 (bottom); three independent preparations were tested and are denoted on the figure as “Sample A”, “Sample B”, and “Sample C”. WCL refers to U937 whole cell lysate used as positive control. At least 25 biological replicates of this purification scheme have been performed and analyzed; the data presented here are representative of the findings. **B** Mean values ± SEM of plaque assay results from three biologically independent EXi-RVFV preparations are shown, quantifying the virions present in the starting material and following the ultracentrifugation spins and sucrose gradient separation. Plaque assays were performed for every single purified exosome preparation throughout our studies, totaling at least 15 biological replicates. A summary of plaque assay results for all the fractions is also presented. The red box highlights the two fractions that were combined to generate EXi-RVFV used in our studies (1.08 g ml-1 and 1.12 g ml-1 fractions). Circles with blue color in the middle represent exosomes and circles with yellow color in the middle represent virions. **C** TEM analysis of purified exosomes is shown. **D** ZetaView analyses of the mean diameter of the EXu and EXi-RVFV populations are presented. Three biological replicates were analyzed. **E** ZetaView measurements of the surface charge (Zeta Potential) for EXu and EXi-RVFV samples are presented. Mean values ± SEM from three biological replicates are shown. **F** ZetaView measurements of the concentration of EXu and EXi-RVFV samples that were matched for total protein content based on BCA are presented. Mean values ± SEM from three biological replicates are shown

We used an optimized sucrose density gradient to allow separation of exosomes and *Bunyavirales*. Exosomes typically have a density range of about 1.13–1.19 g/ml depending on cell type of origin, and bunyaviruses display a density of about 1.21 g/ml [31, 32]. Our optimized sucrose density gradient range consists of five different densities in the range between 1.05 g/ml and 1.25 g/ml (Fig. 1A). We found that both the exosomes originating from uninfected cells and from RVFV-infected cells migrate mainly between the 1.08 g/ml and 1.15 g/ml density fractions, as demonstrated by the presence of the well-established exosome marker CD63 (Fig. 1A). We have performed over 25 biological repeats (fully independent runs) of this purification procedure followed by exosomal marker analysis, demonstrating the robustness and high reproducibility of the procedure. For all our studies, we combined the 1.08 g/ml and 1.12 g/ml density fractions, which contain purified exosomes, into a single preparation. Throughout this study, we refer to this sample of combined 1.08 g/ml and 1.12 g/ml density fractions as EXu for exosomes released from uninfected cells, and EXi-RVFV for exosomes released from RVFV-infected cells. These fractions were selected for combining since they contain the majority of recovered EXu (Fig. 1A), and to match with the EXu control we also combined them for preparation and analysis of the EXi-RVFV. In addition, they are well-separated from the most bottom fraction that contains virions as discussed further below. We have also tested biological repeats of these combined fractions for the presence of the additional well-established exosome markers TSG101, Flotillin-1, and CD9. As anticipated, the purified exosomes are also positive for these additional markers (Fig. 1A, Fig. 6A and B).

We performed plaque assay analysis to verify that EXi-RVFV were efficiently separated from RVFV virions by sucrose density gradient centrifugation (Fig. 1B). While RVFV virions were present in the starting material and the crude exosome pellet, the EXi-RVFV fractions did not contain RVFV virions. Our plaque assay analysis of the remaining fractions from the sucrose density gradient showed the presence of the virus only in the most bottom fraction, consistent with the reported density of 1.21 g/ml for the bunyaviruses (Fig. 1B). These results suggest efficient separation of the exosome fractions that we have used for our studies from virions, or virus like particle (VLPs) that might be present as they would have the same buoyant density as the virions. This conclusion is further confirmed by additional characterizations that we present below, including the results of our CD9 pull down studies that demonstrate specific removal of exosomes from EXi-RVFV completely abolishes their associated biological activities and also that the supernatant left behind after CD9 pull down lacks a signal for either the viral Gn or the viral N protein (Fig. 6). Together, these results confirm the efficiency and specificity of our density gradient procedure.

We also characterized the density gradient-purified exosomes by both transmission electron microscopy (TEM) and ZetaView nanoparticle tracking analysis. The TEM studies demonstrated that we recover intact vesicles through our purification procedure and that there is no difference between the EXu and EXi-RVFV in terms of vesicle morphology and size distributions (Fig. 1C). ZetaView analysis, which allows the study of the vesicles in solution, also demonstrated the presence of intact vesicles in the EXu and EXi-RVFV samples with essentially identical size distributions (Additional file 2: Figure S2), and an average diameter size of about 140 nm for both the EXu controls and EXi-RVFV (Fig. 1D). The average diameter size of the vesicles shown by both the TEM and the ZetaView studies are fully consistent with the reported size range of exosomes [33, 34]. The ZetaView analysis also showed a surface charge of roughly 30 mV for both EXu and EXi-RVFV, corresponding to high stability levels (Fig. 1E). Furthermore, the ZetaView analysis showed that equivalent numbers of EXu and EXi-RVFV vesicles are obtained when they are normalized by the total protein content amounts (Fig. 1F), demonstrating the validity of the protein quantitation approach to normalize exosome amounts between different preparations.

### Sucrose gradient-purified EXi-RVFV contain viral RNA and proteins

**Fig. 2:**
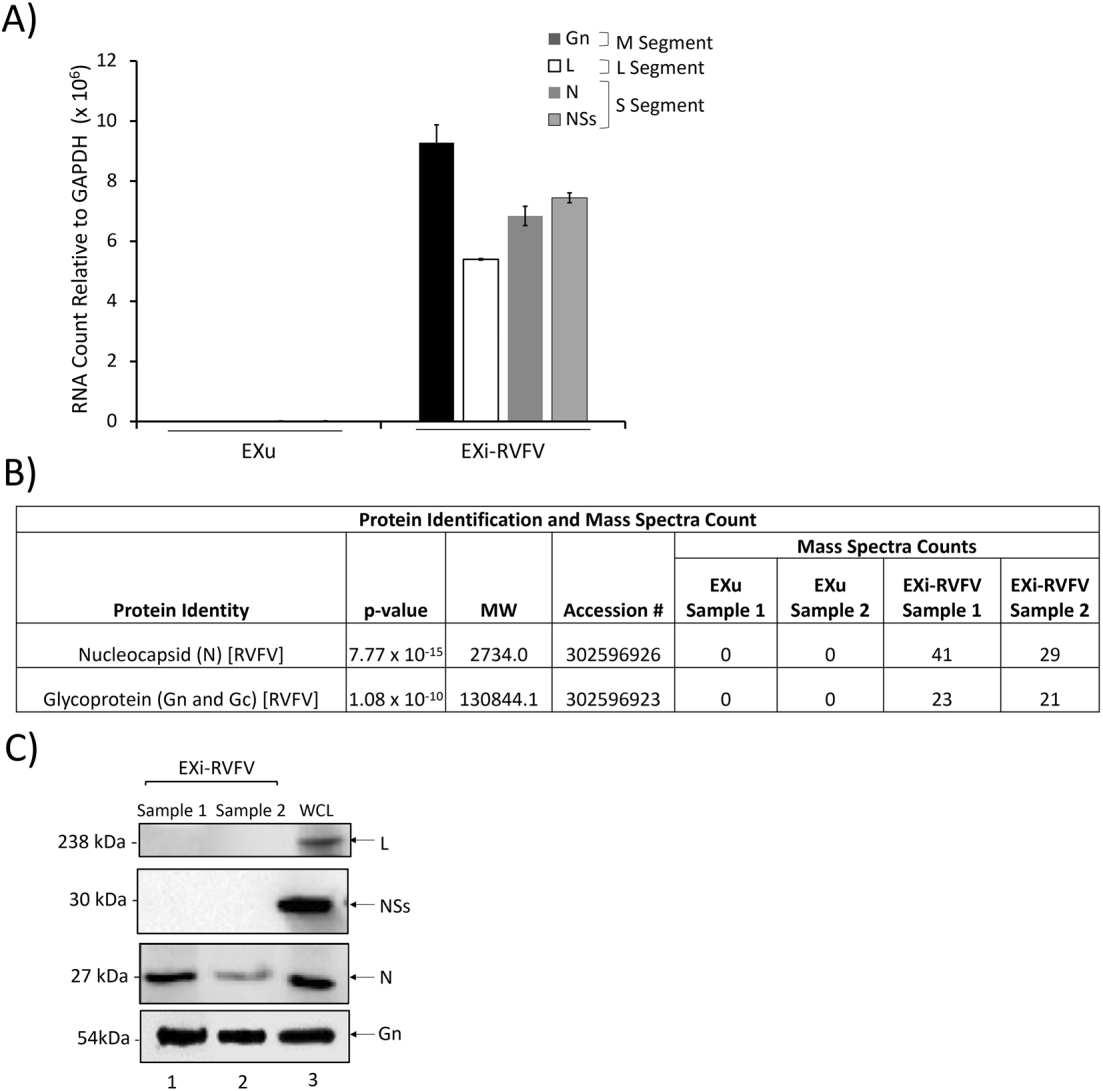
EXi-RVFV Contain Viral RNA and Select Viral Proteins. **A** Total RNA extracts from the EXu and EXi-RVFV samples were subjected to RT-qPCR analysis using RVFV-specific primer pairs. Absolute quantitation of the samples was performed using the cycle threshold (Ct) value relative to the standard curve. Mean values ± SEM from three biological replicates are shown. **B** The results of mass spectrometry analysis of EXi-RVFV to identify associated viral proteins are shown. EXu samples were included as negative control. Mass spectral counts for each identified viral protein is reported. For each sample (EXu or EXi-RVFV), two biological replicates were analyzed. **C** EXi-RVFV samples were analyzed by western blot for the presence of the viral L, NSs, N, and G proteins. WLC corresponds to whole cell lysate preparations from RVFV-infected Vero cells that were included as positive control. Two biological repeat samples (1 and 2) were analyzed

RNA was isolated from EXu and EXi-RVFV, and analyzed by RT-qPCR using primer pairs specific for the three viral genome segments (L, M, and S) to determine whether RNA sequences corresponding to the RVFV genome associate with exosomes. Sequences corresponding to all the viral RNA segments (L, M, S) were present in the EXi-RVFV population, while they were not detected in the negative control EXu sample (Fig. 2A).

High sensitivity and specificity mass spectrometry (MS) analysis was performed on two biological replicates of EXi-RVFV and EXu (as negative control) to analyze the EXi-RVFV for the presence of viral proteins. Two viral proteins, the nucleocapsid protein (N) and the viral glycoprotein (Gn/Gc), were associated with the EXi-RVFV at significant levels, with average spectral counts of 35 and 22 respectively (Fig. 2B). Confirming the MS results, western blot analysis of EXi-RVFV also showed the presence of both the viral N protein and the viral G protein (Fig. 2C). As discussed further below, immunoprecipitated EXi-RVFV obtained by CD9 pull down also gives western signal for the N and G viral proteins, substantiating further the association of these two proteins with the exosomes. Furthermore, the NSs protein or RdRp (L) protein of RVFV were not detected by either MS or western blot analyses (Fig. 2B, C), indicating their absence from the EXi-RVFV. The absence of the L protein in the EXi-RVFV samples corroborates the plaque assay results showing an absence of detectable virions in the EXi-RVFV preparations. These results demonstrate that viral RNA sequences are present in the EXi-RVFV and suggest selective packaging of RVFV proteins into exosomes.

### Pretreatment of cells with EXi-RVFV reduces viral replication during infection

**Fig. 3:**
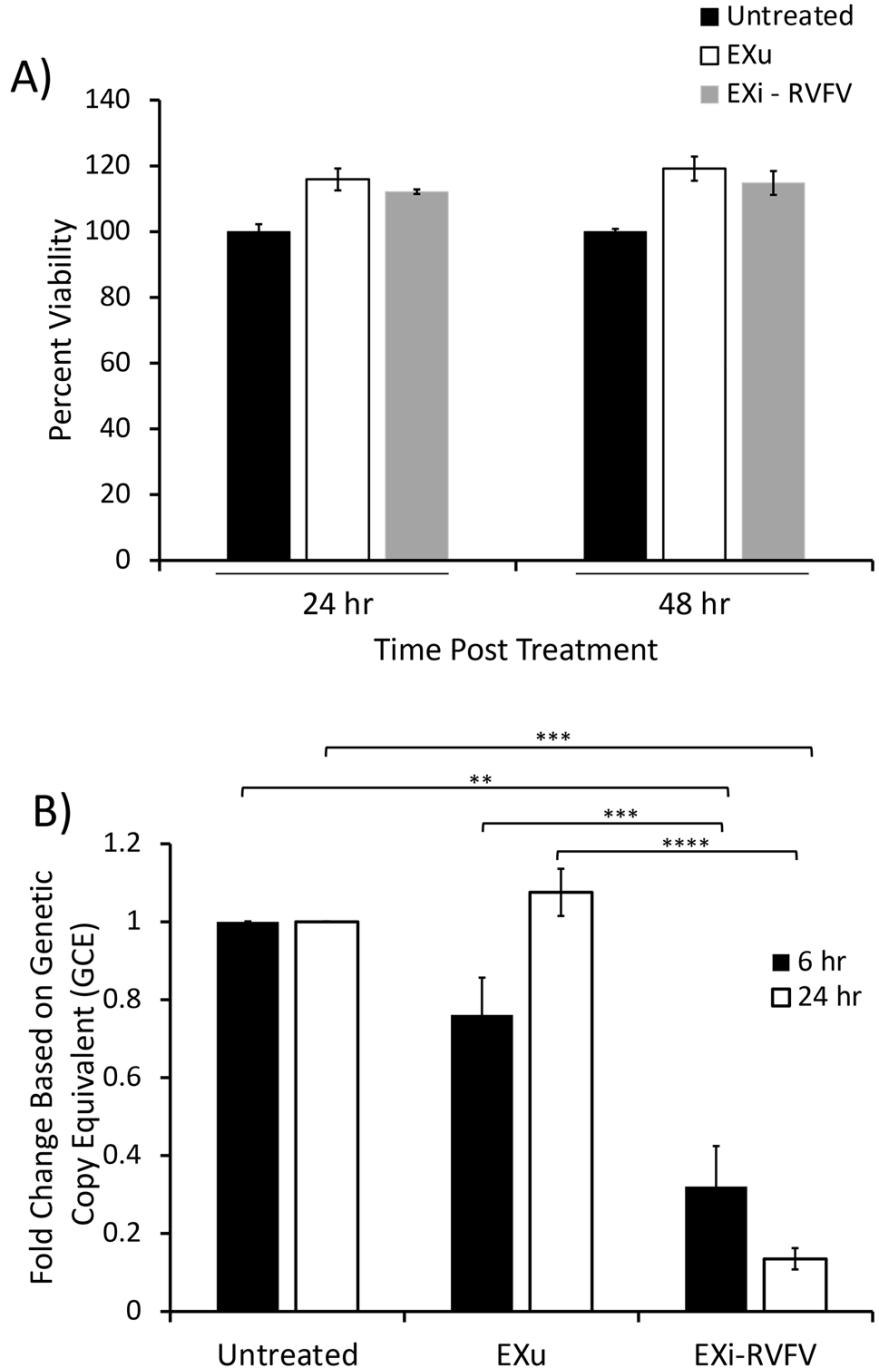
Pre-treatment of Naïve Recipient Human Lung Epithelial Cells with EXi-RVFV Significantly Reduces Viral Replication in the Cells during Subsequent Infection.** A** Naïve HSAECs were treated with either EXu or EXi-RVFV and cell viability was measured using acridine orange/propidium iodide (AO/PI) stain at 24 and 48 h. Mean values ± SEM from three biological replicates are shown. **B** Viral loads in naïve HSAECs that had been pretreated with either EXu or EXi-RVFV and subsequently infected with RVFV were compared based on genetic copy equivalent (RT-qPCR) measurements. Both the 6 h p.i. and the 24 h p.i. time points were analyzed. For each treatment condition and time point assayed, mean values ± SEM from three biological replicates are shown. **P ≤ 0.01; ***P ≤ 0.005; ****P ≤ 0.0005

One of the key features of RVFV pathogenesis is the rapid viral replication resulting in extremely high titers in the target tissue [35]. We analyzed EXi-RVFV for a potential role in modulating viral loads or viral dissemination using Human Small Airway Epithelial Cells (HSAECs), which is an established cell infection model for RVFV [36]. We first verified that exosome treatment did not affect HSAEC viability up to 48 h post treatment (Fig. 3A). Next, we pre-treated HSAECs with either EXi-RVFV, or EXu as control, for 24 h followed by infection with RVFV. At either 6 h or 24 h post-infection (p.i.), intracellular viral loads were determined. Results shown in Fig. 3B demonstrate that while pretreatment with control EXu has no effect on the viral load, pretreating the cells with EXi-RVFV results in a highly significant decrease in intracellular RVFV levels. Thus, a greater than three-fold reduction is observed at 6 h p.i., and an approximately eight-fold reduction is observed at 24 h p.i. (Fig. 3B).

**Fig. 4:**
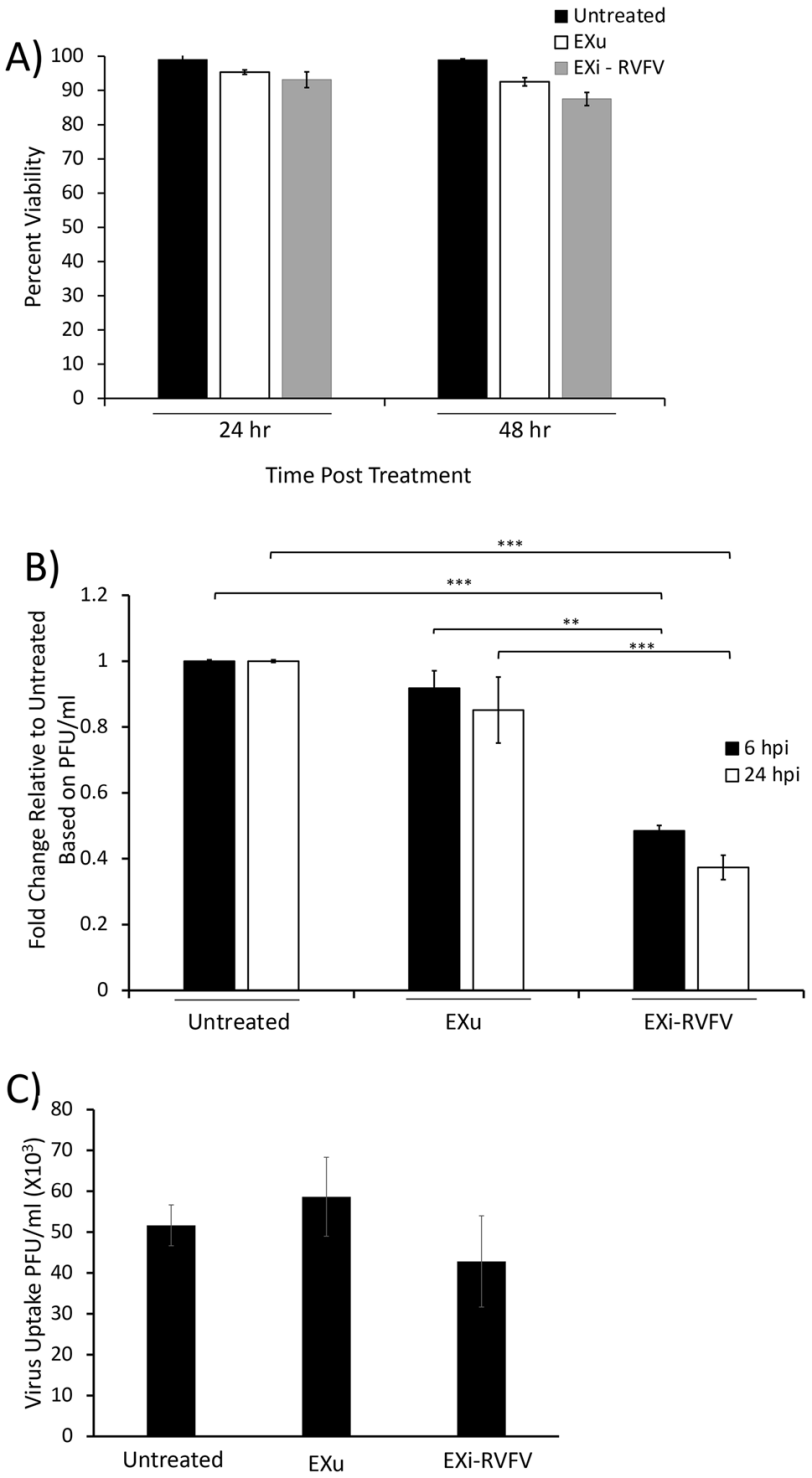
Pre-treatment of Naïve Recipient Human Monocytes with EXi-RVFV Significantly Reduces Viral Replication in the Cells during Subsequent Infection. **A** Naïve U937 cells were treated with either EXu or EXi-RVFV for 24 or 48 h, and cell counts and viability reads were subsequently performed using acridine orange/propidium iodide (AO/PI) staining. For each treatment condition and time point assayed, mean values ± SEM from three biological replicates are shown. **B** Plaque assays were performed to measure viral load in naïve U937 cells that had been pretreated with either EXu or EXi-RVFV and subsequently infected with RVFV. Both the 6 h p.i. and the 24 h pi. time points were analyzed. For each treatment condition and time point assayed, mean values ± SEM from three biological replicates are shown. **C** Plaque assays were performed to measure viral uptake by U937 cells post treatment with either EXu or EXi-RVFV for 24 h and subsequent infection with RVFV. Untreated cells were also included as control. At 1 h p.i., the cells were lysed and intracellular viral loads were determined by plaque assay. For cell lysis, three freeze–thaw cycles were performed in an ethanol-dry ice bath to release the intracellular virus, and quantitative lysis of the cells was verified through microscopic visualization of the sample. Mean values ± SEM from three biological replicates are shown. **P ≤ 0.01; ***P ≤ 0.005

We also performed this study with the human cell line U937 derived from monocytes, which are a RVFV reservoir during infection [37]. Similar to HSAECs, viability of U937 cells was not affected by exosomes at 48 h post treatment (Fig. 4A). To confirm the results obtained by the viability assay, we also measured caspase 3 and caspase 1 activities in response to EXi-RVFV treatment. For these studies, U937 cells were pretreated with either EXi-RVFV, or EXu as control, and incubated for 48 h before analysis. Confirming the cell viability measurements, we observed a lack of either caspase 1 or caspase 3 activation, demonstrating an absence of apoptosis or pyroptosis in EXi-RVFV-treated cells (Additional file 3: Fig. S3). Pre-treatment of the U937 cells with EXi-RVFV resulted in a significant reduction of intracellular viral load at both the 6 h p.i. and the 24 h p.i. (Fig. 4B), in agreement with the results obtained with HSAECs cells. Since the active phase of RVFV replication and transcription occurs within the initial 10 h post entry [16, 38], the early viral load effects seen with EXi-RVFV (6 h p.i.) point to either an effect on viral entry or to effects on viral replication/transcription, or both. To determine whether viral entry was affected after pretreatment with exosomes and subsequent addition of RVFV, the cells were washed twice at 1 h p.i. and then harvested and processed, thus allowing for viral entry but not viral replication. Quantification of the viral RNA indicated that immediately after infection there is no difference in the intracellular viral load between the EXi-RVFV treated cells and either the control untreated but infected cells, or the control EXu treated cells (Fig. 4C), demonstrating that EXi-RVFV function does not impair viral entry into the cell. Collectively, these findings suggest that EXi-RVFV render cells refractory to subsequent infection with RVFV by inhibition of viral replication/transcription.

### EXi-RVFV promote an increase in intracellular interferon-beta (IFN-B) production in naïve recipient cells

**Fig. 5:**
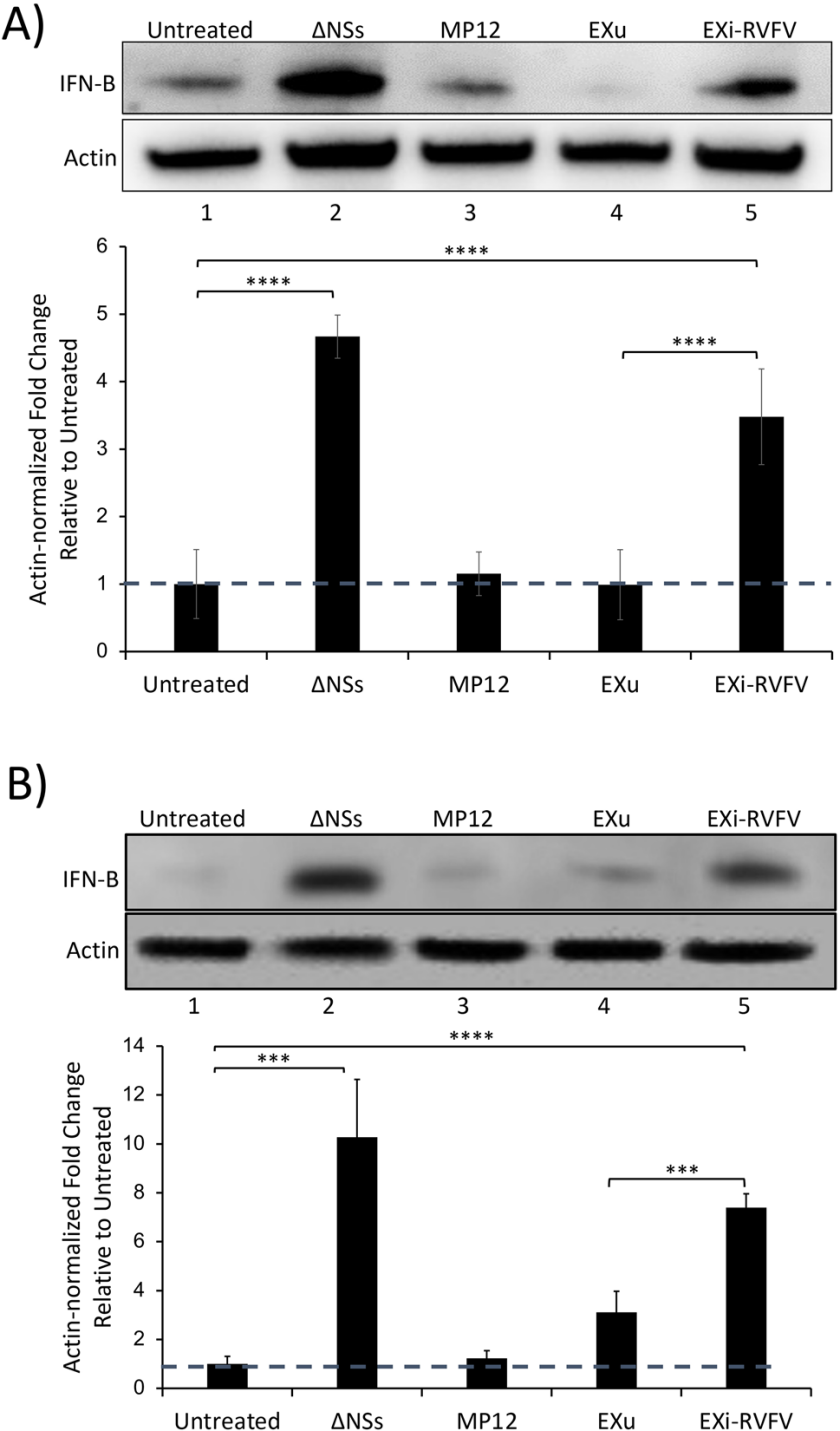
EXi-RVFV Induce Interferon-B in Naïve Recipient Cells. Naïve U937 cells (**A**)**,** or naïve HSAECs (**B**)**,** were either left untreated, or were treated with EXu or EXi-RVFV, and intracellular IFN-B expression was analyzed at 24 h post treatment by western blot analysis of whole cell lysates. Infection with the ΔNSs mutant derivative of the MP12 strain of RVFV was included as positive control, along with infection with the MP12 strain. For each treatment condition, mean values ± SEM from three biological replicates are shown. ***P ≤ 0.005; ****P ≤ 0.0005

Since IFN-B response is the main primary defense against RVFV [12, 39], and EXi-RVFV render recipient cells refractory to subsequent infection with the virus, we analyzed the ability of EXi-RVFV to induce IFN-B in naïve recipient cells. We performed western blot analysis on whole cell lysates to measure IFN-B protein expression 24 h post EXi-RVFV treatment of both U937 cells (Fig. 5A) and HSAECs (Fig. 5B). Infection with a NSs deletion strain of RVFV (ΔNSs) that relieves NSs-dependent repression of IFN-B expression [12] was included as a negative control. As expected, infection with RVFV did not induce IFN-B**,** whereas infection with the ΔNSs strain resulted in a significant activation of IFN-B (Fig. 5A and B). Intriguingly, treatment of naïve human monocytes (U937 cells) with EXi-RVFV also results in a significant increase in IFN-B levels, corresponding to an almost fourfold increase compared to the untreated or EXu-treated cells (Fig. 5A). This degree of activation was similar in magnitude to what is observed by challenging the cells with the ΔNSs mutant strain of RVFV. Similarly, when HSAECs were treated with EXi-RVFV, we observed a significant increase in intracellular IFN-B as compared to control conditions (Fig. 5B).

**Fig. 6:**
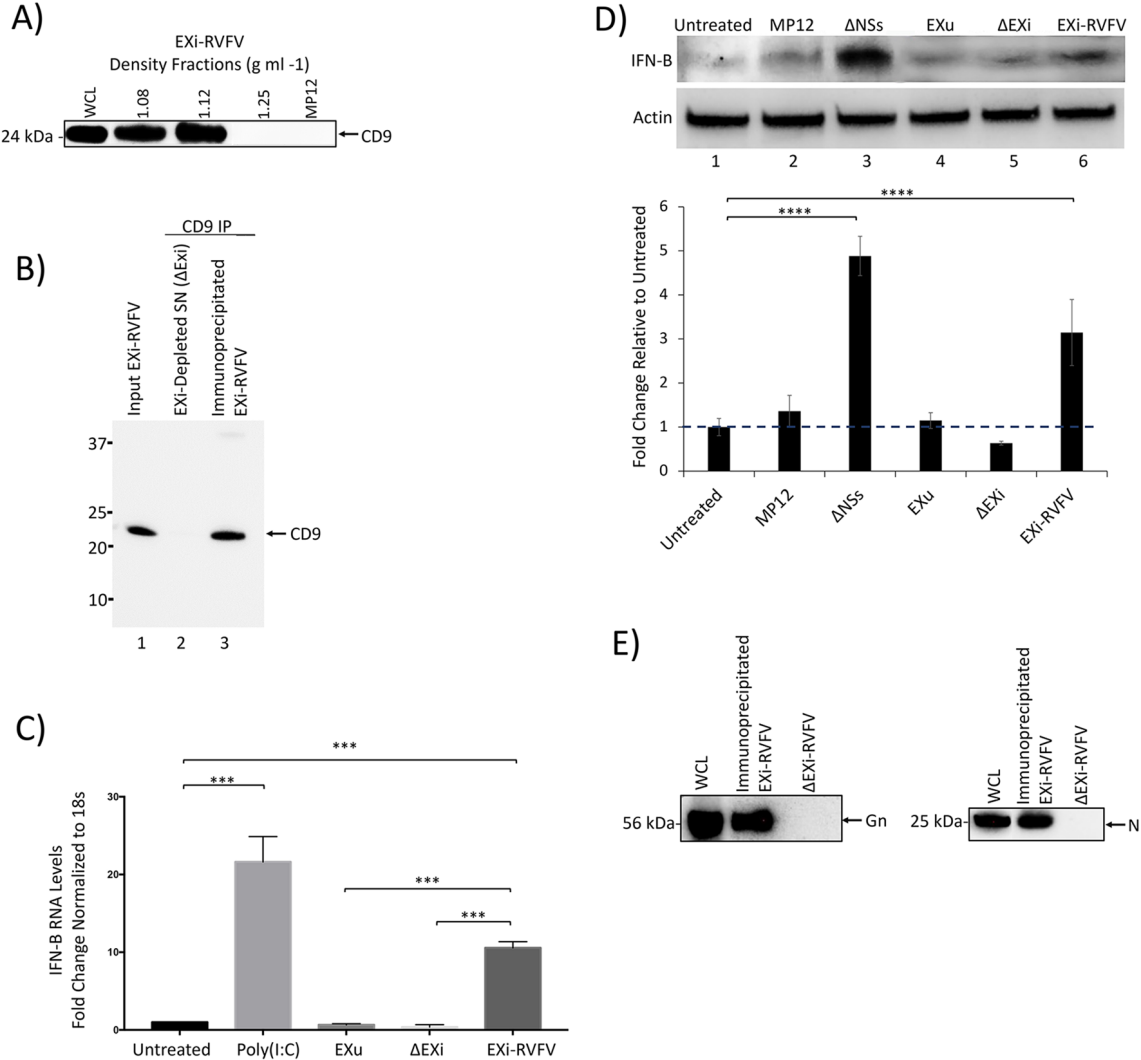
EXi-RVFV Depletion Eliminates Interferon-B Induction in Naïve Recipient Cells.** A** The sucrose density fractions containing EXi-RVFV, the most bottom fraction containing virus (density of 1.25 g ml^−1^), and the working stock of MP12 strain (1.97 × 10^7^ pfu/ml), were analyzed by western blot for the exosome marker CD9. **B** EXi-RVFV samples were subjected to immunoprecipitation using magnetic Dynabeads coated with human anti-CD9 antibody. The efficacy of pull-down was analyzed by anti-CD9 immunoblot of the starting material (Input EXi-RVFV), the immunoprecipitated EXi-RVFV, and the EXi-depleted supernatant that was left behind (ΔEXi). Three biological repeats were performed. A typical western result is shown. **C** Naïve U937 cells were either left untreated, or were subjected to one of the following treatments: i) Poly(I:C); ii) EXu; iii) EXi-RVFV; iv) ΔEXi. The IFN-B expression levels in treated cells were then quantified by RT-qPCR analysis. For each treatment condition, mean value ± SEM from three biological replicates is shown (***P ≤ 0.005). **D** Anti-IFN-B immunoblot analysis was performed to compare IFN-B induction in naïve U937 cells treated with either EXi-RVFV or samples depleted of EXi-RVFV by anti-CD9 IP pulldown (ΔEXi). The intracellular IFN-B expression was analyzed at 24 h post treatment. Untreated cells, cells treated with EXu, or cells infected with either the MP12 strain or the ΔNSs mutant strain of RVFV, were also analyzed side by side. For each treatment condition, mean values ± SEM from three biological replicates are shown in the density bar graph at the bottom. ****P ≤ 0.0005. **E** Western blot analyses of the viral G protein and the viral N protein were performed for immunoprecipitated EXi-RVFV obtained by CD9 pulldown and the resulting supernatant depleted of exosomes (ΔEXi)

As described above, our characterizations of the density gradient fractions indicate lack of virions/VLPs in our EXi-RVFV preparations. Nevertheless, to provide yet another line of evidence that the observed IFN-B effect is due to EXi-RVFV, we subjected the EXi-RVFV preparations to IP pull down of the exosomes using anti-CD9 antibody and analyzed the exosome-depleted supernatant remaining behind for the ability to induce IFN-B. CD9 protein is a well-established and accepted specific marker for exosomes that is not carried by MP12. Our high sensitivity and accuracy MS analysis of purified MP12 virions to profile the associated host proteins has shown the absence of CD9 in MP12 [40]. Furthermore, confirming the MS results, our CD9 western blot analysis of MP12 strain shows the absence of CD9 in this strain (Fig. 6A). This is also demonstrated by CD9 western analysis of the density gradient fractions, showing that while the EXi-RVFV fractions contain CD9, the most bottom fraction that contains virions based on plaque assay (Fig. 1B) lacks any CD9 signal (Fig. 6A). Therefore, CD9 pull down selectively brings down exosomes, and not any MP12 virions or VLPs. Immunoprecipitation with anti-CD9 antibody efficiently removed exosomes from the EXi-RVFV samples (Fig. 6B). The exosome-depleted supernatant remaining behind (designated as ΔEXi) was then tested for IFN-B induction, using both RT-qPCR and western blot assays. The ΔEXi supernatant lacked the ability to activate IFN-B expression in U937 cells and showed the same basal level of expression as the EXu or untreated controls, while the starting EXi-RVFV sample before the pulldown showed a very significant induction of IFN-B expression, giving a greater than tenfold increase in expression levels compared to the EXu and untreated control conditions (Fig. 6C). Similarly, ΔEXi lacked the ability to activate IFN-B production by western blot analysis, showing the same basal levels as the EXu or untreated controls, whereas EXi-RVFV and the ΔNSs RVFV mutant significantly induced IFN-B (Fig. 6D). Furthermore, western analysis of immunoprecipitated EXi-RVFV and ΔEXi for both the viral G protein and the viral N protein show that while immunoprecipitated EXi-RVFV contain both G and N, confirming the results of Fig. 2, the exosome-depleted ΔEXi lacks a signal for either of these viral proteins (Fig. 6E). The lack of either N protein or G protein signal for ΔEXi is consistent with the observed lack of IFN-B activation by ΔEXi and provides further evidence for the absence of biologically significant levels of noninfectious virions within EXi-RVFV. Taken together, our results demonstrate that the observed activation of IFN-B is directly linked to exposure of the cells to the EXi-RVFV.

### EXi-RVFV induces IFN-B through activation of the RIG-I pathway

**Fig. 7:**
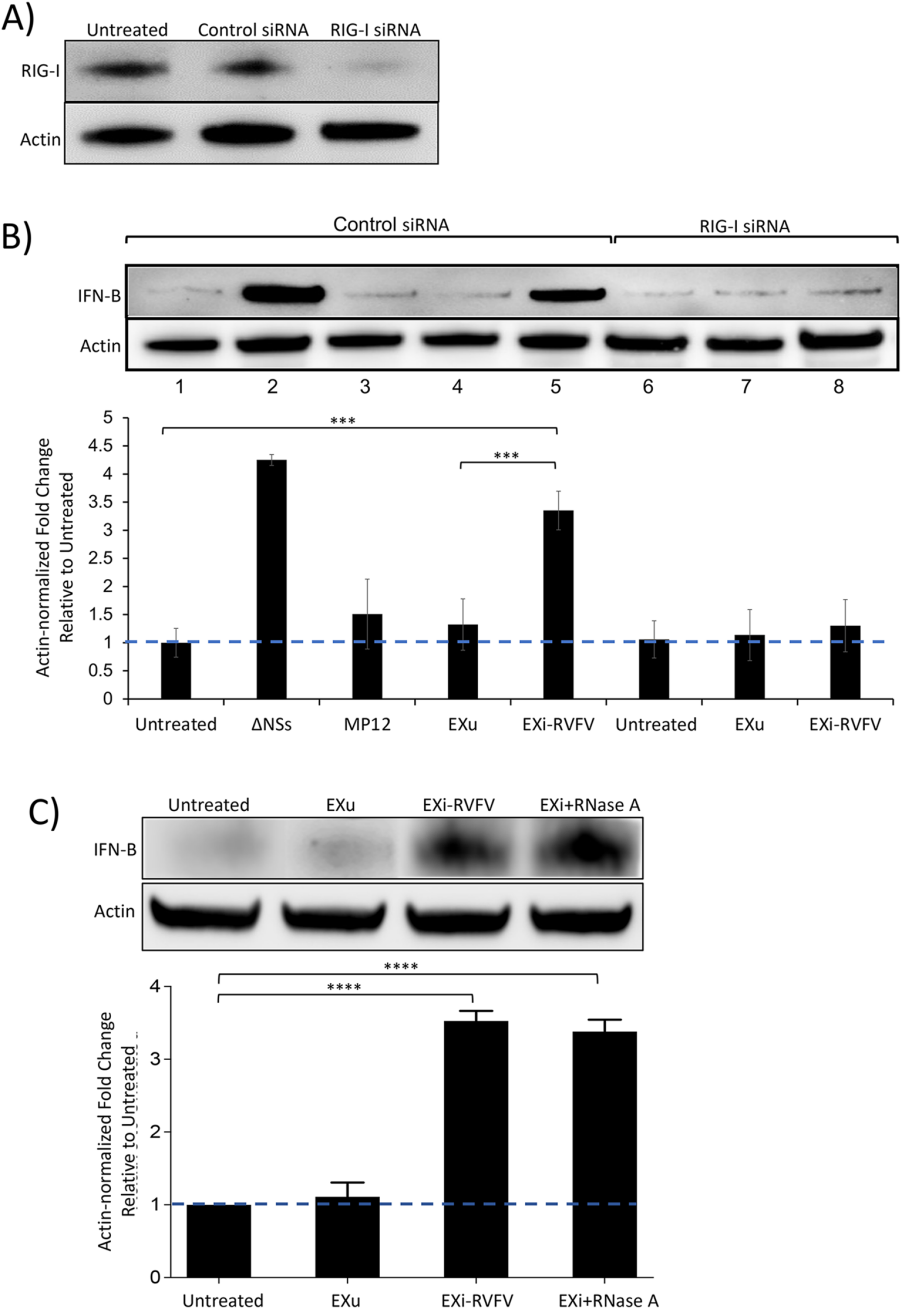
EXi-RVFV Induction of Interferon-B Is Mediated by RIG-I Activation and Is Impervious to RNase Treatment of the Exosomes. **A** U937 cells were transfected with RIG-I siRNA (Santa Cruz) and RIG-I expression was measured at 24 h p.i. by western blot analysis. Cells that were either left untransfected or transfected with control scrambled siRNA (Santa Cruz) were also included as controls. **B** U937 cells were transfected with either a scrambled control siRNA (Santa Cruz) or with RIG-I siRNA (Santa Cruz) 24 h prior to any further treatment. Subsequently, the cells were split to generate the following treatment conditions: (i) Uninfected (control); (ii) Infection with the MP12-L-V5, NSs-Flag strain of RVFV; (iii) Infection with the ΔNSs derivative of MP12 strain (positive control); (iv) Treatment with EXu without any infection; (v) Treatment with EXi-RVFV without any infection. Whole cell lysates were prepared 24 h post treatment or post infection, and analyzed by western blot for IFN-B expression. **C** Naïve U937 cells were either left untreated or were treated with EXu, or EXi-RVFV, or RNase-treated EXI-RVFV (EXi-RVFV + RNase), and intracellular IFN-B expression was analyzed at 24 h post treatment by western blot analysis of whole cell lysates. For each treatment condition, mean values ± SEM from three biological replicates are shown. ***P ≤ 0.005; ****P ≤ 0.0005

The retinoic acid-inducible gene I (RIG-I, encoded by the gene DDX58) is a well-characterized member of the RIG-I-like receptor (RLR) family of pattern recognition receptors (PRRs) that senses viral RNA and activates signaling pathways that lead to the expression and secretion of type I IFNs, including IFN-B and other proinflammatory cytokines [41]. The purified RNA genome of RVFV has been shown to activate IFN-B in a RIG-I-dependent manner [42]. Therefore, given that viral RNA sequences are carried by EXi-RVFV, we investigated the potential contribution of RIG-I to the EXi-RVFV dependent activation of IFN-B. siRNA knockdown of RIG-I in human monocytes resulted in roughly a 90% reduction in RIG-I levels (Fig. 7A). EXi-RVFV induction of IFN-B was absent in naïve recipient monocytes in which RIG-I was knocked down while no reduction in IFN-B expression was observed in the cells treated with the control siRNA, (Fig. 7B), indicating that the IFN-B activation by EXi-RVFV is dependent on RIG-I activity.

To determine if the exosomal RNAs that induce IFN-B through RIG-I in recipient cells are packaged within the vesicles or if instead they associate with the exosomes on the outside surface, we analyzed the ability of RNase A-treated EXi-RVFV to activate IFN-B expression. ZetaView analysis of RNase A-treated vesicles verified that they have remained intact, as expected. Following RNase A treatment of EXi-RVFV, or EXu as control, to degrade any RNAs that may be associating with the outside of the vesicles, RNase was removed by dialysis against PBS buffer and naïve U937 cells were treated with the exosome preparations. Western blot analysis shows that there is no difference between the RNase-treated EXi-RVFV and untreated EXi-RVFV in the ability to induce significant IFN-B production in recipient cells as compared to the control conditions (Fig. 7C). Thus, our results demonstrate that the RNA species responsible for IFN-B activation through the RIG-I pathway are packaged within the EXi-RVFV**.**

### EXi-RVFV induction of IFN-B leads to activation of autophagy

**Fig. 8:**
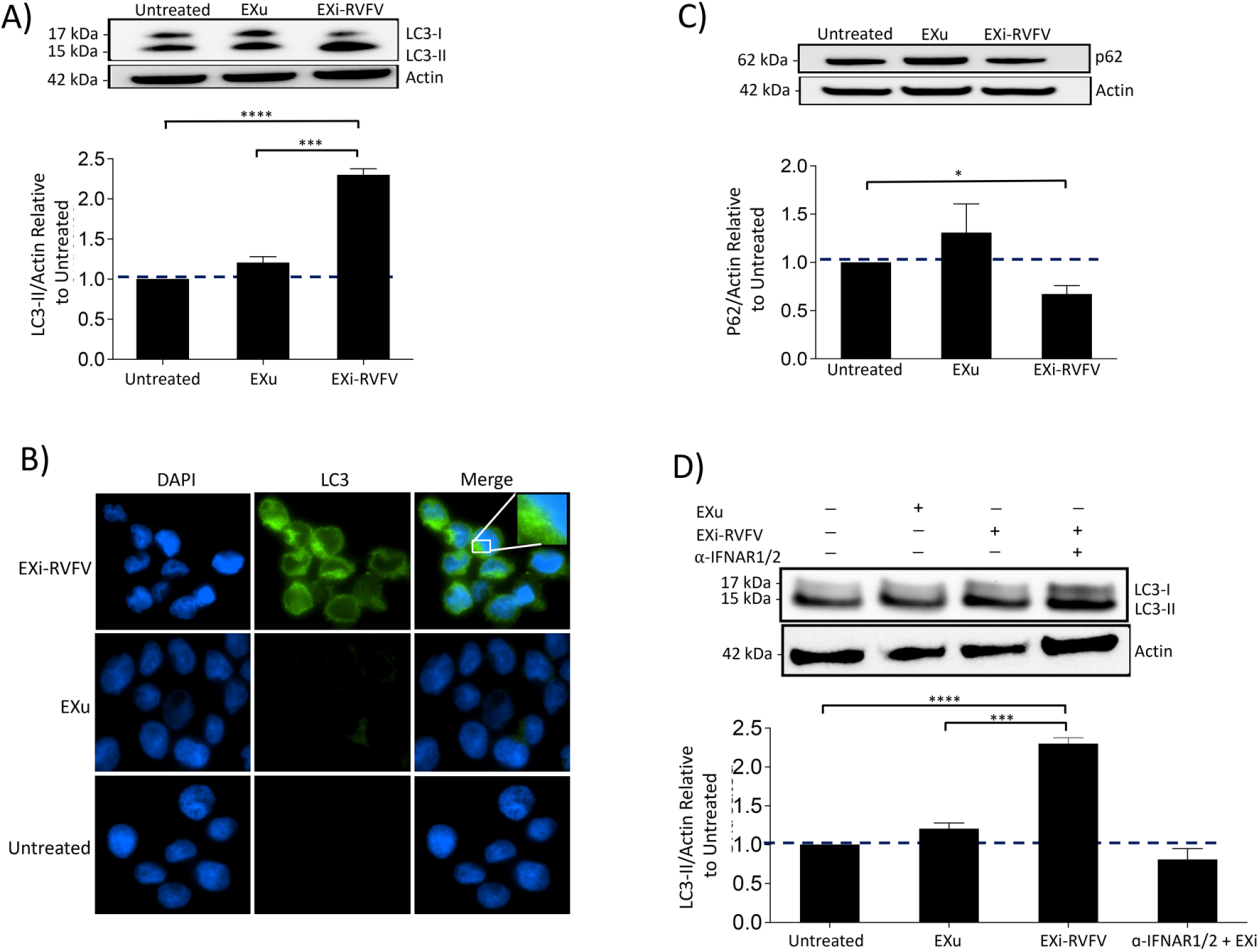
Treatment of Naïve Recipient Human Monocytes with EXi-RVFV Activates Autophagy through the Induction of IFN-B. **A** Naïve U937 cells were either left untreated, or were treated with EXi-RVFV or EXu for 24 h, and whole cell lysates matched for total protein levels were subjected to western blot analysis for LC3. Mean values ± SEM for three biological replicates is shown. **B** Immunofluorescence microscopy analysis of LC3 expression was performed for U937 cells that were either left untreated, or were treated with EXi-RVFV or EXu for 24 h, followed by LC3 antibody staining. Green signal represents LC3 and blue signal corresponds to DAPI staining of the nuclei. **C** Naïve U937 cells were either left untreated, or were treated with EXi-RVFV or EXu for 24 h, and whole cell lysates matched for total protein levels were subjected to western blot analysis to measure p62 levels. Mean values ± SEM from two biological replicates is shown. **D** Naïve U937 cell were pre-treated with anti-IFNAR2 antibody for 4 h and subsequently were treated with EXi-RVFV for 24 h. Naïve U937 cells without anti-IFNAR1/2 antibody treatment but treated with either EXi-RVFV or EXu were also included to serve as control conditions. Western blot was performed for LC3-II analysis. Mean values ± SEM from two biological replicates are shown. *P ≤ 0.03; **P ≤ 0.01; ***P ≤ 0.005; ****P ≤ 0.0005

Autophagy is activated in response to RVFV infection [43]. Furthermore, IFN-B can activate autophagy in a concentration and time dependent manner [44]. Consequently, we analyzed whether the EXi-RVFV treatment of naïve cells results in activation of autophagy similar to infection with RVFV, and if so whether such activation is dependent on the EXi-RVFV induction of IFN-B that we have observed. We performed several assays for analysis of changes in autophagy. Western blot analysis of whole cell lysates demonstrated that EXi-RVFV treatment of naïve U937 cells leads to a significant increase in formation of LC3II as compared to untreated cells or cell treated with the EXu control, suggesting activation of autophagy (Fig. 8A). Consistent with these results, immunofluorescence microscopy studies show that naïve U937 cells treated with EXi-RVFV show a dramatic increase in LC3 puncta formation compared to either the untreated cells or cell treated with EXu (Fig. 8B). We also analyzed changes in p62 levels, to evaluate whether the observed increase in autophagy is an indirect consequence of a decrease in autophagic flux or if it is rather indicative of increased autophagosome formation. Western blot analysis demonstrated that p62 expression is actually reduced in EXi-RVFV treatment compared to the control conditions (Fig. 8C), indicating an increase in autophagic flux. These results indicate that EXi-RVFV treatment leads to activation of autophagy through both increased autophagosome formation and increased autophagy degradation activity.

We next analyzed whether the EXi-RVFV activation of autophagy is dependent on the observed activation of IFN-B by these exosomes. Following treatment with anti-IFNAR antibody to block IFN activity, the ability of EXi-RVFV to induce autophagy was examined by LC3 western blot analysis. The results show that blocking IFN activity completely inhibits activation of autophagy by the EXi-RVFV (Fig. 8D). Taken together, our results demonstrate that the EXi-RVFV activate autophagy through induction of IFN-B.

## Discussion

Exosomes released from human cells play a key role in the body response to a variety of diseases, including viral infection [20, 45, 46]. We previously described the initial characterization of exosomes derived from cells that are resistant to RVFV infection and demonstrated the association of RVFV RNA genome segments and specific RVFV proteins with these vesicles [28]. In the current study, we have developed a a step-wise sucrose gradient procedure to purify EXi-RVFV and characterized their content. Furthermore, we have peformed mechansitic studies of EXi-RVFV interaction with naïve recipient cells and their effect on RVFV pathogenesis.

The EXi-RVFV vesicles are intact and show appropriate density range and size range for exosomes, and are positive for several exosomal markers. We have also shown a lack of detectable virions by various assays and criteria, including plaque assay analysis shown in Fig. 1B, the lack of a western signal for the viral L protein in the EXi-RVFV (Fig. 2C), and the lack of western signal for either the Gn protein or N protein of the virus in what remains behind after selective depletion of exosomes from the EXi-RVFV by CD9 immunoprecipitaion (Fig. 6E). In addition, our CD9 pull down studies of the purified EXi-RVFV (Fig. 6) show that the effects observed from treatment of naïve recipient cells with these vesicle preparations are due to the function of the exosomes. The CD9 protein, which serves as a specific marker for exosomes, is not carried by MP12, based on both the state of the art MS analysis of purified MP12 virions to profile the associated host proteins [40] and the western blot analysis that we have presented here. This is consistent with the fact that CD9 is present on the plasma membrane (from which late endosomal membranes and therefore exosomes derive) whereas it is absent from the golgi [47–49] through which RVFV trafficking and assembly takes place [50, 51]. The reported characterization of RVFV VLPs [52] provides yet further support for our findings that the observed biological effects is caused by the EXi-RVFV and not VLPs/noninfectious virions. On sucrose density gradients, RVFV VLPs migrate to a fraction corresponding to about 50–55% sucrose, which is also fully consistent with the reported density of around 1.21 g/ml for bunyaviruses. This sucrose percentage is very different than the sucrose percentages of the exosome fractions that we have collected for our studies (21% sucrose and 28% sucrose respectively).

Consistent with our previous findings for resistant clone exosomes [28], the EXi-RVFV carry sequences corresponding to the three RVFV RNA genome segments. In addition, they carry the viral N and Gn/Gc proteins but lack the L polymerase (RdRp) or the NSs protein. This suggests that in our previous study those resistant clone exosomes that carried N but lacked any form of NSs may be better representatives of exosomes released during regular infection. Furthermore, since we do not observe induction of apoptosis by EXi-RVFV in this study, the resistant clone exosomes that did not induce apoptosis in recipient cells may better represent natural infection. Most significantly, the present study shows that EXi-RVFV serve a protective role for the host, which is in contrast to many other viral infections in which exosomes derived from virus-infected cells aid in viral propagation or are detrimental to their target recipient cells [46, 53]. Pretreatment of human monocytes or epithelial cells with EXi-RVFV prior to infection with RVFV drastically reduces intracellular viral levels without affecting host cell viability. This effect is not due to a decrease in viral entry but rather to the inhibition of the active phase of viral replication/transcription.

The protective role of EXi-RVFV can be attributed to the induction of IFN-B in the recipient cells before the virus reaches them. The NSs protein of RVFV is known to suppress the induction of the antiviral type 1 interferon response, thus increasing virulence [39]. IFN response has been shown to be a key component of the host defense against RVFV and to be effective as potential RVFV therapy [54, 55]. Furthermore, there are vaccine candidates based on inducing IFN-A/B responses [56]. Therefore, the ability of the EXi-RVFV to upregulate IFN-B production might be exploited in the development of novel preventative/therapeutic advances.

We have also analyzed the mechanism by which the EXi-RVFV induce activation of IFN-B, showing its dependency on the RIG-I activation pathway, which specifically recognizes viral-derived RNAs to regulate the immune response to viruses such as influenza A virus, hepatitis C virus, Sendai virus, and flaviviruses [57–60]. An uncapped 5′ end and extended dsRNA secondary structures within the genome of several RNA viruses play a pivotal role in RIG-I stimulation [61–63]. RIG-I preferentially binds to relatively short (< 300 bp) uncapped (5′-ppp end) or double stranded RNA (dsRNA), panhandle or bulge/loop structures [61, 64, 65], although small 5′-hydroxylated, 3′-phosphorylated RNAs produced by the RNase L cleavage of the hepatitis C virus RNA can also efficiently activate RIG-I [66] and so do short structured RNAs transcribed from the Kaposi’s sarcoma-associated herpesvirus genome [67]. Previous work indicated that the presence of a 5′ triphosphate and an undefined dsRNA sequence withint the RVFV genome can trigger RIG-I [68]. Since sequences corresponding to all three RVFV genome segments are present in EXi-RVFV, it is plausible that one or more of the exosome-associated RVFV RNAs might trigger the RIG-I dependent IFN-B expression. In accordance with this hypothesis, exosomal transfer of short 5′-triphosphate RNAs synthesized from the Epstein–Barr virus (EBV) genome trigger antiviral immunity in dendritic cells [69]. Furthermore, the proposed activation of interferon response by EXi-RVFV through exosome-associated viral RNA is reminiscent of the report by Dreux and colleagues describing that the viral RNA present within exosomes released from HCV-infected cells can induce IFN-A release from uninfected plasmacytoid DCs (pDCs) through involvement of the TLR-7 pathway [70]. Similarly, RNA transfer by exosomes released from cells infected with the lymphocytic choriomeningitis virus (LCMV) activates IFN-A production by human pDCs through a TLR7-dependent mechanism [71].

Induction of autophagy is one of the cellular effects associated with IFN-B activity [44]. Furthermore, autophagy is activated as part of the host response to RVFV infection, resulting in a significant reduction of viral burden [43]. Consistent with these findings, we had predicted previously that activation of autophagy may occur in response to exosomes of infection origin, leading to our observed increased clearance of pathogens that may enter the cell subsequently [72]. Our results presented here are in line with this prediction, demonstrating that autophagy is indeed activated in response to EXi-RVFV, as demonstrated by both LC3 activation and increased autophagy flux. Furthermore, we have shown that this activation is dependent on the EXi-RVFV induction of IFN-B. To our knowledge, our studies are the first demonstration of RIG-I-dependent activation of IFN-B by exosomes released from virally infected cells, and also the first demonstration of exosome-mediated induction of autophagy through IFN-B activation. In future studies, we plan to profile the RNA species packaged in the EXi-RVFV as part of efforts to identify the RNA sequences responsible for the observed RIG-I activation. We also plan to determine whether the EXi-RVFV can protect against other viral infections for which IFN and autophagy responses play an important role as well.

**Fig. 9:**
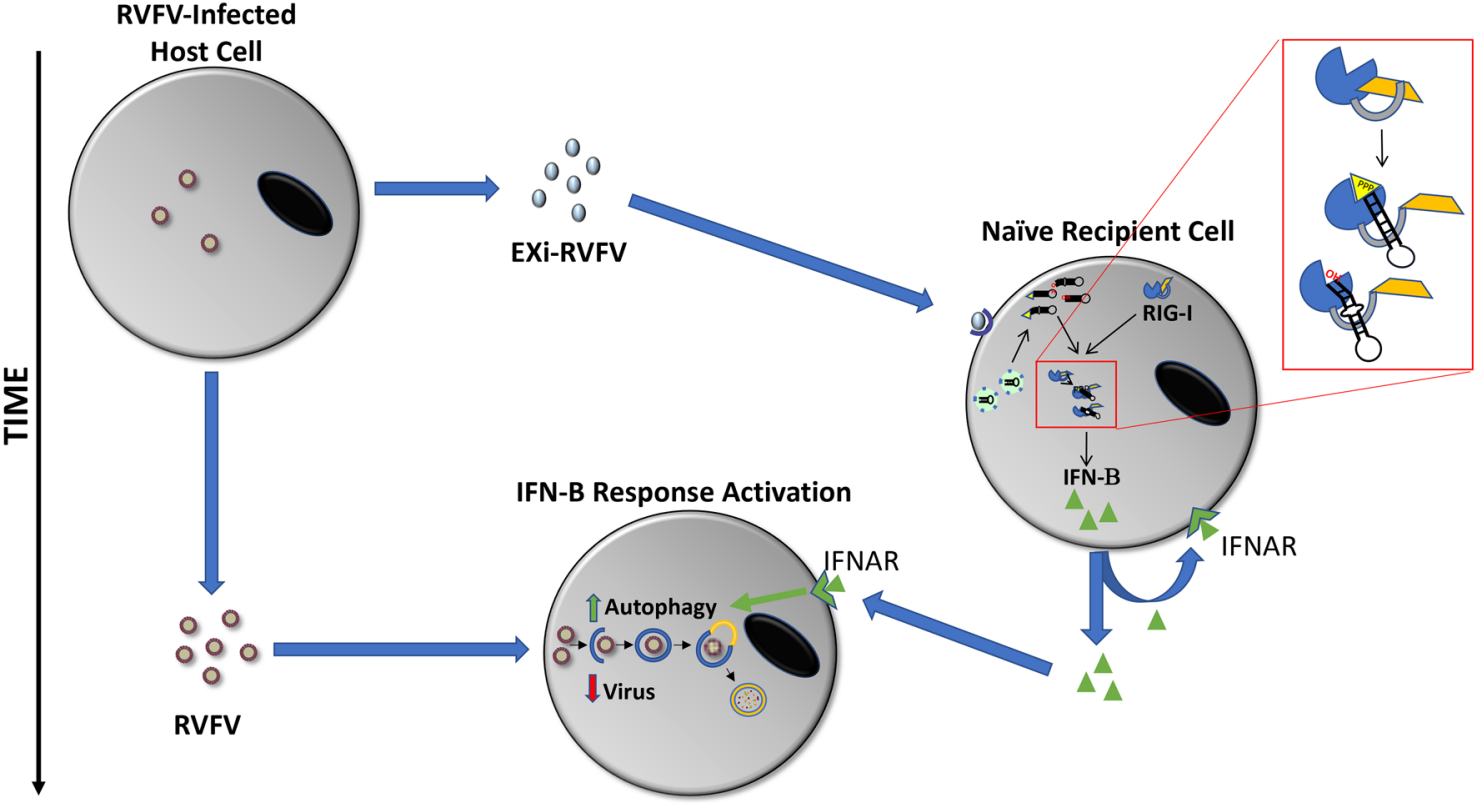
Proposed Model of Exosomal Function During RVFV Infection. EXi-RVFV released by infected cells travel to local and distant target naïve cells. Following entry, viral RNA genome carried by EXi-RVFV are released first, and specific viral RNA sequence(s) that form appropriate dsRNA panhandle structures induce RIG-I. The activation of RIG-I leads to induction of IFN-B that is subsequently released to elicit both autocrine and paracrine IFN responses that involves activation of autophagy. Increased autophagy leads to encapsulation of virus particles that could reach and enter the cell at a later stage, eliminating them through autolysosomes to result in a significant decrease in viral replication, and therefore propagation

In summary, our results suggest that exosomes from RVFV infected cells may alter the dynamics of the neighboring cells and potentially interfere with the disease pathology by providing protection to their target cells. In this model, the EXi-RVFV released from the infected cells reach naïve target immune cells prior to their encounter with the virus, resulting in production of RIG-induced IFN response in these cells and subsequent activation of autophagy before they become infected. Consequently, viral replication in these cells is strongly inhibited when the virus subsequently reaches them (Fig. 9). It is of great interest to examine whether the exosome-mediated IFN-B and autophagy activation effects reported here also apply for other pathogenic RNA viruses, such as SARS-CoV-2 virus that is the cause of the ongoing worldwide COVID-19 pandemic. Intriguingly, in this connection, misregulation of type I IFN activity has been associated with severe cases of COVID-19 [73, 74]. Furthermore, exosomes have been successfully used as a drug delivery system for nucleic acids and proteins with a high therapeutic efficacy and low levels of toxicity and immunogenicity [75, 76]. Given these features, the continuous need for safe and efficient RVFV countermeasures for human and animal use, and the ability of the EXi-RVFV to activate IFN response, it is worthwhile to explore the potential of EXi-RVFV to aid in development of desirable vaccine/therapeutic platforms. The availability of established animals models of RVFV infection, such as the mouse and non-human primate (NHP) models [77, 78], will help in taking the next steps in this direction.

## Supplementary Information

**Additional file 1: Figure S1.** Viability of Vero cells Post Infection with MP12. The viabilities of uninfected Vero cells and the same batch of cells infected with MP12 were measured at 36 h post infection. Mean values ± SEM from three biological replicates are shown.

**Additional file 2: Figure S2.** Size Distributions of EXu and EXi-RVFV Vesicles. Representative ZetaView analysis of size distributions for EXu and EXi-RVFV are presented.

**Additional file 3: Figure S3.** EXi-RVF Treatment Does Not Induce Apoptosis or Pyroptosis.** A** U937 cells were either left untreated or were treated with EXu or EXi-RVFV for 48 h, and caspase 1 activity was subsequently measured. Purified active human caspase 1 was included as positive control. For each treatment condition, mean values ± SEM from three biological replicates are shown. Statistical analysis was performed for comparison of purified caspase-1 treatment with untreated control. **B** U937 cells were either left untreated or were treated with EXu or EXi-RVFV for 48 h, and caspase 3 activity was subsequently measured. Irradiated U937 cells were included as positive control. For each treatment condition, mean values ± SEM from three biological replicates are shown. Statistical analysis was performed for comparing irradiated cells with untreated control. **P ≤ 0.01.

## Data Availability

The datasets generated during and/or analyzed during the current study are available in the PeptideAtlas repository (accession number PASS01683), https://db.systemsbiology.net/sbeams/cgi/PeptideAtlas/PASS_View?identifier=PASS01683.
