# Supplementary figures and images for: Exosomes originating from infection with the cytoplasmic single-stranded RNA virus Rift Valley fever virus (RVFV) protect recipient cells by inducing RIG-I mediated IFN-B response that leads to activation of autophagy
Source: Cell Biosci. 2021 Dec 25;11:220. doi: 10.1186/s13578-021-00732-z (PMC8710069; doi:10.1186/s13578-021-00732-z)

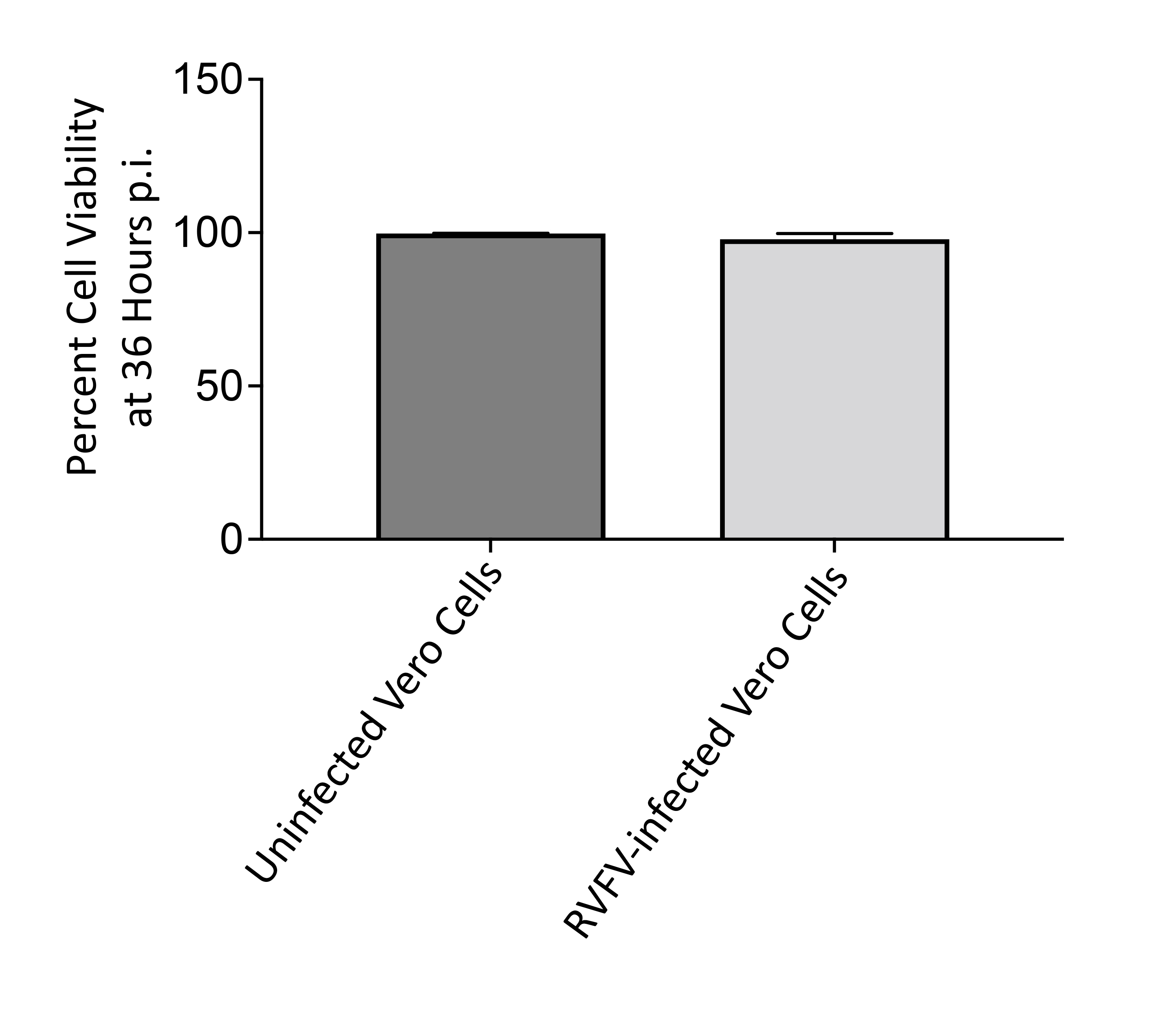

Supplement: Supplementary file 1 — Additional file 1: Figure S1. Viability of Vero cells Post Infection with MP12. The viabilities of uninfected Vero cells and the same batch of cells infected with MP12 were measured at 36 h post infection. Mean values ± SEM from three biological replicates are shown. [file 13578_2021_732_MOESM1_ESM.jpg]

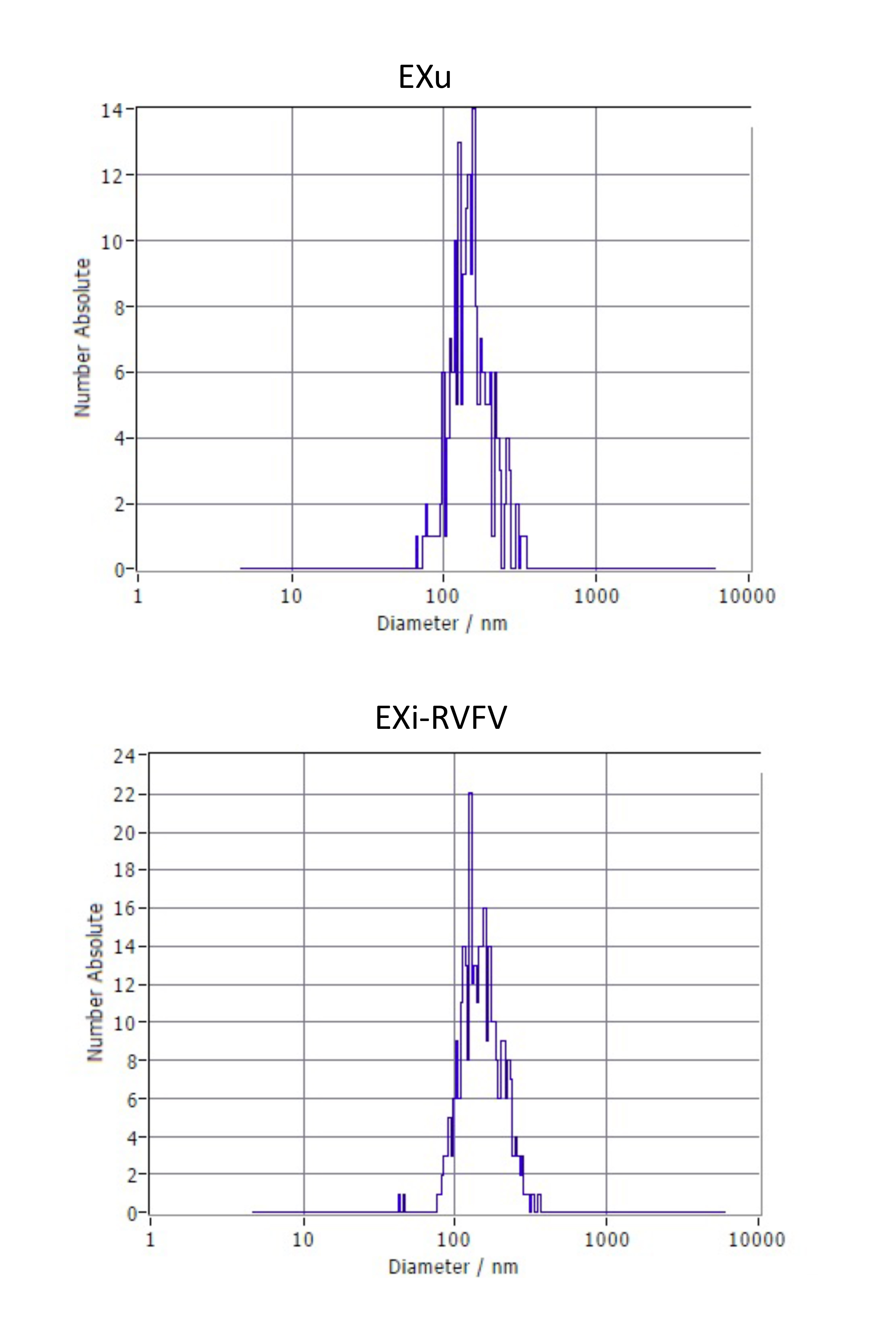

Supplement: Supplementary file 2 — Additional file 2: Figure S2. Size Distributions of EXu and EXi-RVFV Vesicles. Representative ZetaView analysis of size distributions for EXu and EXi-RVFV are presented. [file 13578_2021_732_MOESM2_ESM.jpg]

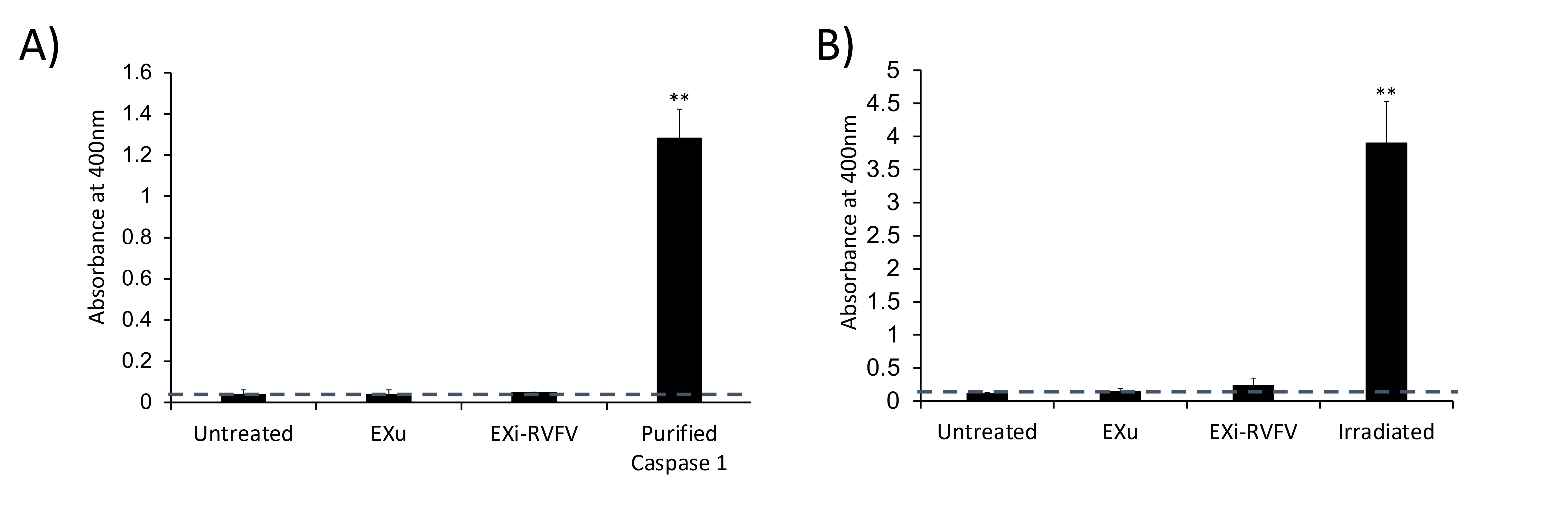

Supplement: Supplementary file 3 — Additional file 3: Figure S3. EXi-RVF Treatment Does Not Induce Apoptosis or Pyroptosis. A U937 cells were either left untreated or were treated with EXu or EXi-RVFV for 48 h, and caspase 1 activity was subsequently measured. Purified active human caspase 1 was included as positive control. For each treatment condition, mean values ± SEM from three biological replicates are shown. Statistical analysis was performed for comparison of purified caspase-1 treatment with untreated control. B U937 cells were either left untreated or were treated with EXu or EXi-RVFV for 48 h, and caspase 3 activity was subsequently measured. Irradiated U937 cells were included as positive control. For each treatment condition, mean values ± SEM from three biological replicates are shown. Statistical analysis was performed for comparing irradiated cells with untreated control. **P ≤ 0.01. [file 13578_2021_732_MOESM3_ESM.jpg]
